# Analysis of α-Dystroglycan/LG Domain Binding Modes: Investigating Protein Motifs That Regulate the Affinity of Isolated LG Domains

**DOI:** 10.3389/fmolb.2019.00018

**Published:** 2019-03-29

**Authors:** Christopher E. Dempsey, Maria Giulia Bigotti, Josephine C. Adams, Andrea Brancaccio

**Affiliations:** ^1^School of Biochemistry, University of Bristol, Bristol, United Kingdom; ^2^Istituto di Chimica del Riconoscimento Molecolare - CNR, Università Cattolica del Sacro Cuore, Rome, Italy

**Keywords:** dystroglycan, laminin globular (LG) domains, binding affinities, protein modeling, protein structure

## Abstract

Dystroglycan (DG) is an adhesion complex that links the cytoskeleton to the surrounding extracellular matrix in skeletal muscle and a wide variety of other tissues. It is composed of a highly glycosylated extracellular α-DG associated noncovalently with a transmembrane β-DG whose cytodomain interacts with dystrophin and its isoforms. Alpha-dystroglycan (α-DG) binds tightly and in a calcium-dependent fashion to multiple extracellular proteins and proteoglycans, each of which harbors at least one, or, more frequently, tandem arrays of laminin-globular (LG) domains. Considerable biochemical and structural work has accumulated on the α-DG-binding LG domains, highlighting a significant heterogeneity in ligand-binding properties of domains from different proteins as well as between single and multiple LG domains within the same protein. Here we review biochemical, structural, and functional information on the LG domains reported to bind α-dystroglycan. In addition, we have incorporated bioinformatics and modeling to explore whether specific motifs responsible for α-dystroglycan recognition can be identified within isolated LG domains. In particular, we analyzed the LG domains of slits and agrin as well as those of paradigmatic α-DG non-binders such as laminin-α3. While some stretches of basic residues may be important, no universally conserved motifs could be identified. However, the data confirm that the coordinated calcium atom within the LG domain is needed to establish an interaction with the sugars of α-DG, although it appears that this alone is insufficient to mediate significant α-DG binding. We develop a scenario involving different binding modes of a single LG domain unit, or tandemly repeated units, with α-DG. A variability of binding modes might be important to generate a range of affinities to allow physiological regulation of this interaction, reflecting its crucial biological importance.

## Introduction

**Figure 1:**
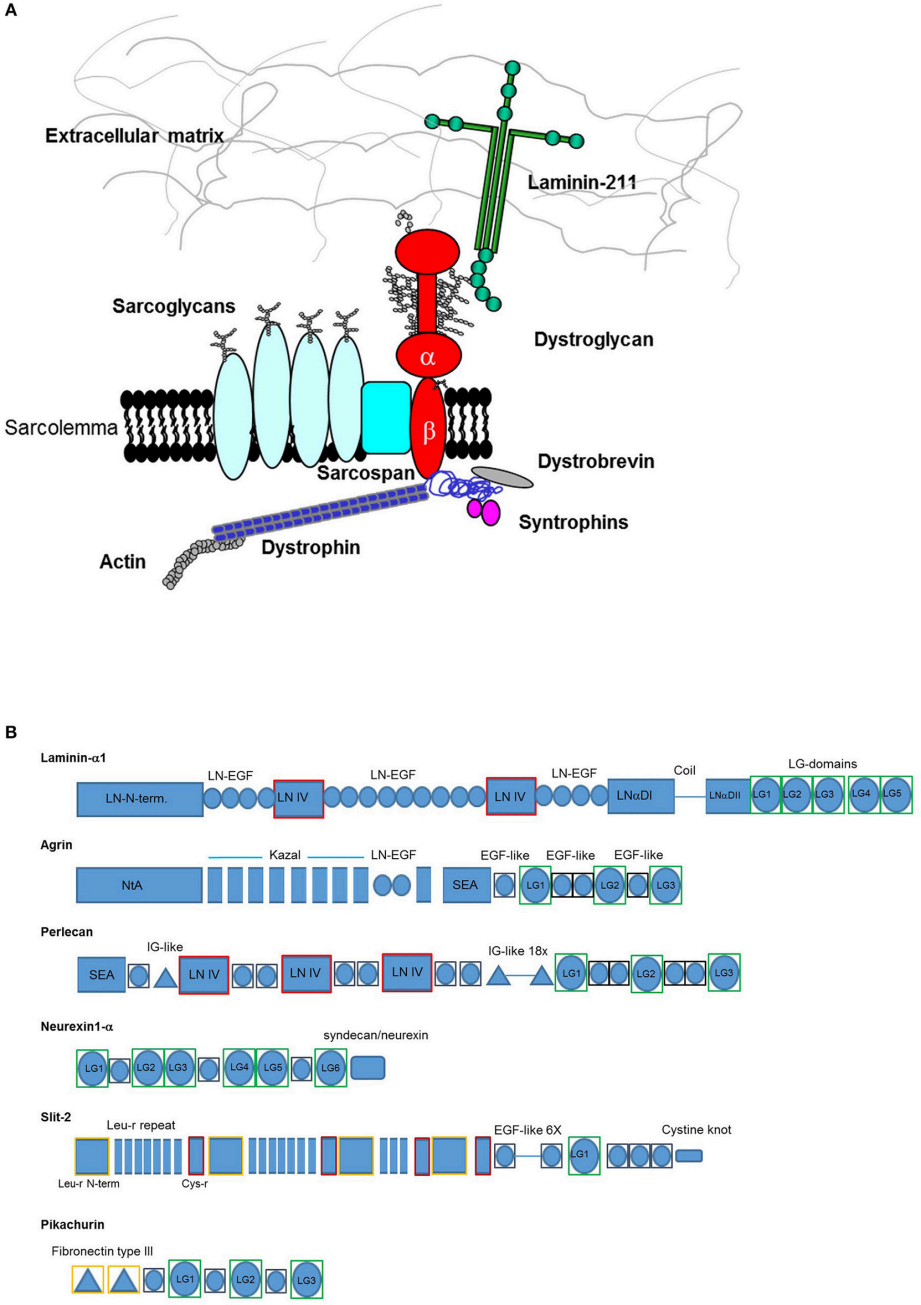
**(A)** The dystrophin-glycoprotein complex (DGC). Dystroglycan is embedded within the membrane as a pivotal member of the complex establishing multiple contacts with extracellular, transmembrane, and intracellular partners. In skeletal muscle it creates a “molecular bridge” between cells and the surrounding tissues offering stability upon muscle contraction. **(B)** Domain architecture of representative α-dystroglycan binding partners. LG domains representing α-DG-binding sites are boxed in green. Each domain has been assessed using InterProScan 5.2 (https://www.ebi.ac.uk/interpro/) database as described in the Supplementary section. Codes: LN-N-term. (Laminin, N-terminal), LN-EGF (Laminin-type epidermal growth factor-like), LN IV (Laminin IV domain), LNαDI (Laminin alpha, domain I), LNDII (Laminin Domain II), NtA (N-terminal of agrin), SEA (Sperm protein, Enterokinase and Agrin), EGF-like (Epidermal growth factor-like), IG-like (Immunoglobulin-like), Leu-r (leucine-rich), Cys-r (cysteine-rich). Not to scale.

In the tissues of multicellular animals, different cell types establish intercellular molecular contacts (junctions) as well as cellular-extracellular ones. Accordingly, connections established between the extracellular matrix (ECM) and appropriate adhesion complexes at the cell surface support the functional morphology and physiology of different tissues during development and in adult animals. A well-conserved array of ECM proteins, cell adhesion receptors and ECM proteases has been identified in animals (Özbek et al., 2010; Hynes, 2012). In this context, dystroglycan is probably the major non-integrin adhesion complex for the formation of molecular contacts that stabilize the interface between cell membranes in skeletal muscle and the specialized ECM surrounding them (i.e., basement membranes) (Adams and Brancaccio, 2015) (see Figure 1A).

Dystroglycan (DG) is composed of two subunits, α-DG which is extracellular and highly glycosylated, and the transmembrane β-DG, whose cytoplasmic domain links the whole adhesion complex to dystrophin and the actin cytoskeleton in skeletal muscle. In addition to skeletal and cardiac muscles, DG has a wide tissue distribution and interacts with utrophin and dystrophin isoforms within the cytoplasm in a variety of tissues, such as the central and peripheral nervous system, lung and kidney (Barresi and Campbell, 2006).

In striking contrast with the integrin family of cell-ECM adhesion receptors, there is only one DG gene (*DAG1*), and no physiologically relevant alternative splicing has been reported so far. Therefore, different α-DG binding affinities for various extracellular ligands seem to depend on the degree of α-DG glycosylation, which might vary in different tissues (McDearmon et al., 2006; Sciandra et al., 2013). In fact, α-DG interacts with members of the laminin family of ECM glycoproteins and several other ECM-associated binding partners through its ability to recognize the so-called laminin-globular (LG) domains (see Figure 1B for a summary of the domain architecture of the binding proteins discussed here). LG domains were originally identified within laminins (Beck et al., 1990) but can be found in several proteins and proteoglycans located in the ECM as well as in a variety of otherwise diverse proteins (Talts et al., 1998). The LG domains that bind α-DG likely represent a distinct subset within the entire LG-domain family and are expected to share common structural features that allow them to recognize α-DG. However, sequence analysis demonstrates a limited degree of homology amongst different LG domains, and it is possible that other members of the LG domain family remain to be identified (Rudenko et al., 2001).

Although other proteins and proteoglycans harboring LG domains (for example, thrombospondins, Adams and Lawler, 2011) might be additional α-DG binders, the focus of this Review is on the established and physiologically relevant molecular partners whose binding behaviors have been tested both *in vivo* and *in vitro*. We examine LG domains demonstrated experimentally to bind α-DG for potential common structural features and compare these domains with LG domains that do not bind α-DG *in vitro*.

Laminins and certain other LG-containing binding partners have high, or very high (K_d_'s within the low nanomolar range) binding affinities for α-DG (Sciandra et al., 2013). The interaction depends on recognition of O-linked sugar moieties that protrude from the central mucin-like domain of α-DG. Multiple analyses from different groups have led to an increased understanding of the glycosylation moieties of O-mannosylated α-dystroglycan, whose complex structure also includes ribitol-phosphate, a newly identified glycosylation unit in mammals (for further details see recent reviews by Manya and Endo, 2017; Sheikh et al., 2017; Kanagawa and Toda, 2018).

Like legume lectins as well as animal galectins, with which they share a similar fold (Rudenko et al., 2001), the LG domains establish crucial protein-sugar interactions to stabilize sarcolemma and other plasma membranes. In this regard, it has recently been shown (Briggs et al., 2016) that the recombinant LG4-LG5 pair of murine laminin-α2 can be crystallized in combination with a polysaccharide that is found in α-DG and belongs to its glycan-repeated scaffold (Willer et al., 2014); this important study also demonstrates the crucial role of a coordinated calcium cation for binding. The disaccharide portion of the sugar molecule identified in the structure is coordinated only to the LG4 domain (Briggs et al., 2016), and any additional contribution of protein-protein interactions in the DG/LG binding, possibly involving portions of the *core protein* of DG, has not so far been confirmed (Bozic et al., 2004). A point of curiosity is that, while many LG-containing proteins include multiple LG domains, a few, such as the slits, contain only a single domain. It is not clear whether this distinction reflects a different mode of binding.

Although in the past 20 years considerable biochemical and structural data (see Table 1) have been collected on laminins (the prototypical LG-containing DG binding partners) and on several other DG binding partners, several key questions concerning dystroglycan-LG binding remain unanswered. For example, is it possible to identify common molecular characteristics (ideally short linear motifs) that would define the propensity to bind α-DG in this domain family? On the other hand, are there some identifiable structural features shared by *non-binding* LG-domains? These questions could have important biological implications, and to review them we have combined two separate lines of analysis. Firstly, we consider the general features of LG domains and re-evaluate the available information on the interaction of α-dystroglycan to its binding partners that share LG domains; secondly, we have carried out homology modeling of LG domains of unknown structure, with a specific focus on two paradigmatic cases, namely the LG domains of human slit and agrin. The modeling results are reported in the paragraphs corresponding to the different binding partners of DG in the sections below, and the methods employed are described in the section “Methods for sequence analysis and homology modeling” of the Supplementary Material.

**Table 1:** LG-containing human proteins and proteoglycans with emphasis on those known to bind α-dystroglycan.

**Protein (α chains for heterotrimeric αβγ laminin)**	**Code name**	**NCBI accession code**	**Length (aa)**	**LGs (aa)**	**DG binding LGs**	**Notes**	**Literature**	**PDB**
Laminin-α1 (present in laminin-111 and laminin-121)	LNA1	P25391	3,075	LG1(181)-LG2(177)-LG3(188)-LG4(173)-LG5(181)	LG4-5 *(so-called domain E3)* **LG4**	Binding is calcium dependent Particularly dependent on basic residues within the RKR, RAR, and KDR motifs in murine LN's LG4	Andac et al., 1999 Talts et al., 1999 Harrison et al., 2007	***murine*** **LG4-5: 2JD4 (LG4)**
Laminin-α2 (present in laminin-211, laminin-221 and laminin-213)	LNA2	P24043	3,122	LG1(184)-LG2(182)-LG3(185)-LG4(172)-LG5(181)	LG1-3 LG4-5 **LG3 (weak)**	Binding is calcium dependent LG4-5 affinity is 4-fold higher than LG4-5 from LNA1 (murine LN) Affinity is dependent on the basic stretch RRKRRQ in murine LG3, whereas in human the stretch is RRKRRR. Mutations to Ala within the motif reduce the affinity (Talts and Timpl, 1999)	Talts et al., 1998 Hohenester et al., 1999 Talts et al., 1999 Tisi et al., 2000 Wizemann et al., 2003 Carafoli et al., 2009 Briggs et al., 2016	***murine*** **LG1-3: 2WJS (LG3)** *murine* LG4-5: 1DYK/1OKQ ***murine*** **LG5: 1QU0** murine LG4-5: 5IK4,5IK5,5IK7,5IK8 (5IK5 and 5IK8, in complex with a single glucuronic acid-b1,3-xylose disaccharide repeat)
Laminin-α3 (present in aminin-332, laminin-311 and laminin-321)	LNA3	Q16787	3,333	LG1(202)-LG2(163)-LG3(161)-LG4(165)-LG5(174)	Not binding	From sequence comparison, predicted not to bind calcium (Timpl et al., 2000) Tested on native laminin-5 preparations (Ferletta et al., 2003; Kikkawa et al., 2004) not on recombinantly expressed domains	Timpl et al. (2000), Ferletta et al. (2003) *α-dystroglycan from rat Schwannoma cells* (Kikkawa et al., 2004) *α-dystroglycan from mouse GD 25 cells*	- PDB not available
Laminin-α4 (present in laminin-411, laminin-421 and laminin-423)	LNA4	Q16363	1,823	LG1(203)-LG2(180)-LG3(169)-LG4(172)-LG5(174)	LG1-3 LG4-5	Lower affinity than the corresponding modules from LNA1 and LNA2 - From sequence comparison, predicted not to bind calcium (Timpl et al., 2000)	Talts et al., 2000	- PDB not available
Laminin-α5 (present in laminin-511, laminin-521 and laminin-523)	LNA5	O15230	3,695	LG1(194)-LG2(175)-LG3(169)-LG4(174)-LG5(173)	LG4-5 **LG4**	Lower affinity than LG4-5 from α1 or α2 Multiple basic amino acid residues in the putative loop regions are involved synergistically in the α-dystroglycan binding by the LG4 module -From sequence comparison, predicted not to bind calcium (Timpl et al., 2000). However, Yu and Talts (2003) observe an EDTA-dependence in LG4-5 binding	Shimizu et al., 1999 Ferletta et al., 2003 Yu and Talts, 2003, Ido et al., 2004 Kikkawa et al., 2004	- PDB not available
Agrin	AGR	O00468	2,067	LG1(177)-LG2(184)-LG3(182)	LG1-2	LG3 does not bind DG. LG1 might represent the best binder in the LG1-2 tandem. Affinity is influenced by a splice site (A: KSRK) on LG2. The variant with KSRK binds less strongly. LG2 is not alone sufficient for high-affinity binding	Campanelli et al., 1996, Gesemann et al., 1996, Hopf and Hoch, 1996, O'Toole et al., 1996, Gesemann et al., 1998, Stetefeld et al., 2004	*Gallus gallus* LG3: 1Q56, 1PZ7, and 1PZ8/1PZ9 (B0, B11 e B8 alternatively spliced forms)
Perlecan	PRL	P98160	4,391	LG1(186)-LG2(181)-LG3(189)	LG1-3 *(so-called domain V)* LG1-2 LG2-3	Only tandem arrays can bind. No binding, or very weak binding, with isolated domains	Friedrich et al., 1999 Talts et al., 1999 Le et al., 2011	***human*** **LG3: 3SH4/3SH5**
Pikachurin	PKC	Q63HQ2	1,017	LG1(179)-LG2(180)-LG3(180)	LG1-3 LG2-3	It is not known if isolated domains can bind	Sato et al., 2008 Kanagawa et al., 2010 Omori et al., 2012	
Neurexin1α	NRX1A	Q9ULB1	1,477	LG1(188)-LG2(183)-LG3(193)-LG4(174)-LG5(176)-LG6(169)	LG1-6 **LG2** **LG6**	There is a plethora of splicing isoforms. Usually, the forms with no inserts bind better to α-DG	Sugita et al., 2001 Sheckler et al., 2006, Chen et al., 2011	*Bos taurus* LG2-6: 3QCW/3R05 *Bos taurus* LG2: 2H0B
Neurexin2α	NRX2A	Q9P2S2	1,712	LG1(179)-LG2(198)-LG3(194)-LG4(173)-LG5(176)-LG6(209)	?			
Neurexin3α	NRX3A	Q9Y4C0	1,643	LG1(176)-LG2(183)-LG3(193)-LG4(173)-LG5(176)-LG6(171)	?			
Neurexin1β	NRX1B	P58400	442	LG1(169)	**LG1**	It is exactly the same as LG6 of Neurexin1α	Rudenko et al., 1999 Shen et al., 2008	*Rattus norvegicus* LG1: 1C4R/2R1D
Neurexin2β	NRX2B	P58401	666	LG1(209)	?			
Neurexin3β	NRX3B	Q9HDB5	637	LG1(171)	?			
Slit-1	SLT1	O75093	1,534	LG1(174)	?	The KVR motif is NOT conserved		
Slit-2	SLT2	O94813	1,529	LG1(174)	**LG1**	A double mutation of two basic residues (KVR to AVA) abolishes binding. EDTA abolishes binding as well	Wright et al., 2012	
Slit-3	SLT3	NP_001258875	1,530	LG1(175)	?	The KVR motif is conserved		

*The list includes the laminin-α3 chain (reported not to bind DG). α-DG binding has not yet been confirmed for the two paralogs of Slit-2*.

*In bold: LG domains which are able to bind α-DG in an isolated fashion, and potential templates used for computational modeling. Laminin-α3 chain sequence refers to the isoform 3A*.

## General Features of LG Domains

**Figure 2:**
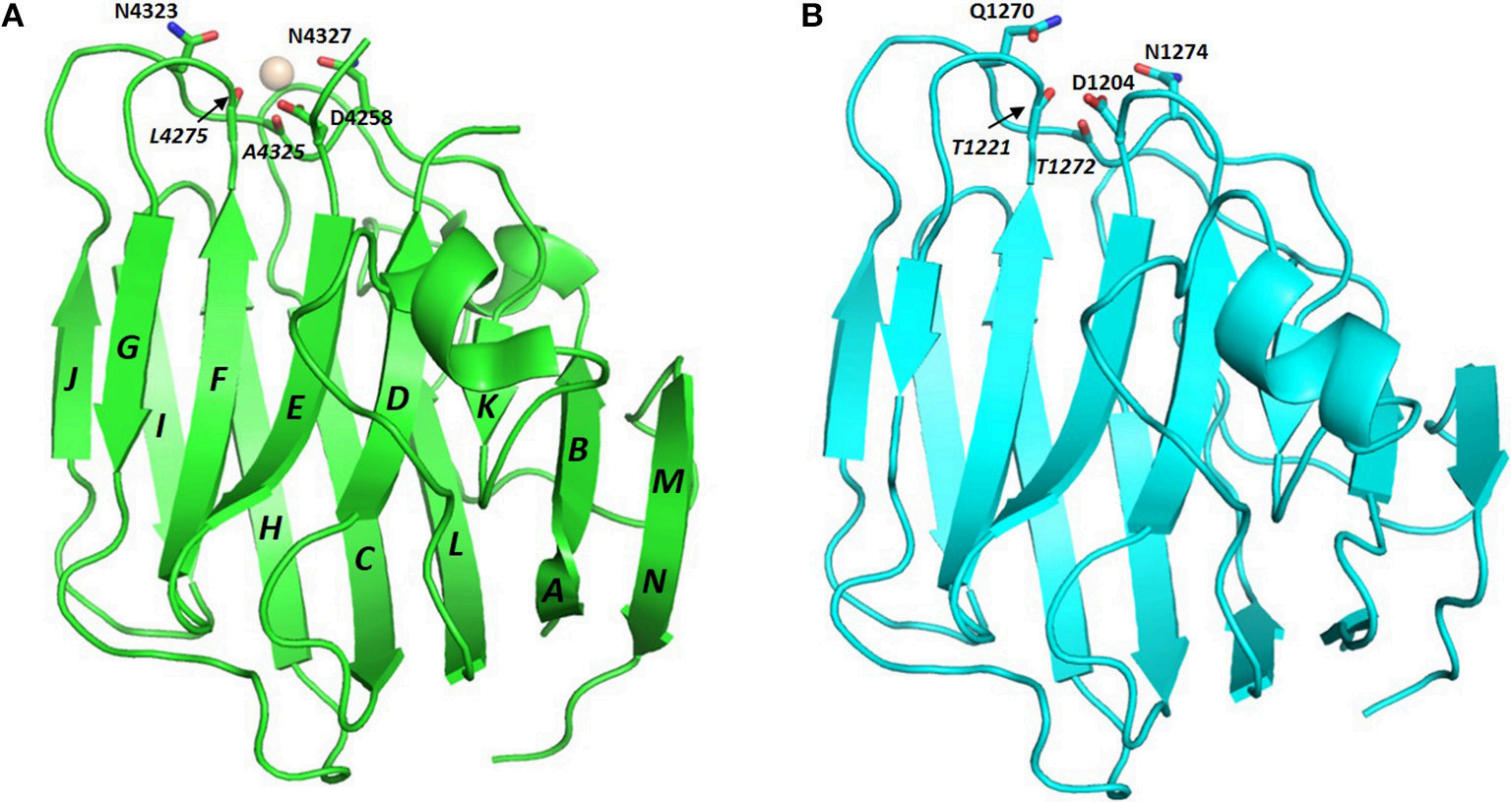
Crystal structure of perlecan LG3 domain. β-strands are annotated according to the scheme of Le et al. (2011). In this representation **(A)** strands M and N are “fused” and a short sequence in the D-E loop is missing density in the crystal structure. The Ca^2+^ ion in the perlecan LG3 Ca^2+^ binding site (ligand residues are highlighted) is shown as a sphere. **(B)** homology model of a slit-2 LG domain constructed on the perlecan LG3 structure template. The slit-2 model produces a potential Ca^2+^-binding domain with a very similar arrangement of backbone and side chain ligands for Ca^2+^ as in the LG3 domain, suggesting that the slit-2 LG domain is likely to bind Ca^2+^. See **Figure 8** for additional interpretation of slit LG domain homology models.

LG domains have a globular/spherical shape and are commonly composed of 160–180 amino acids, mostly organized as a jelly-roll sandwich with two antiparallel seven-stranded β sheets (Timpl et al., 2000; Le et al., 2011). For example, the structure of human perlecan LG3 has been solved both in calcium-bound (PDB: 3SH5) and apo form (PDB: 3SH4) (Le et al., 2011), and comprises 14 β-strands (from A to N) and 2 α-helices (Figure 2). The specific β-strand order, as found in perlecan LG3, JIHCLAN and GFEDKBM (in the two opposing β-sheets, respectively, see Figure 2) applies broadly to all the LG domains whose 3D structures have been resolved, although in some case the strands at the edges of the sheets are not well-defined. Thus, human perlecan LG3 structure is used as a reference in this Review, that mainly focuses on human LG domains. It should be noted that the two α-helices following the B and K strand in perlecan LG3 are not uniformly found in other LG domains of known structure.

**Figure 3:**
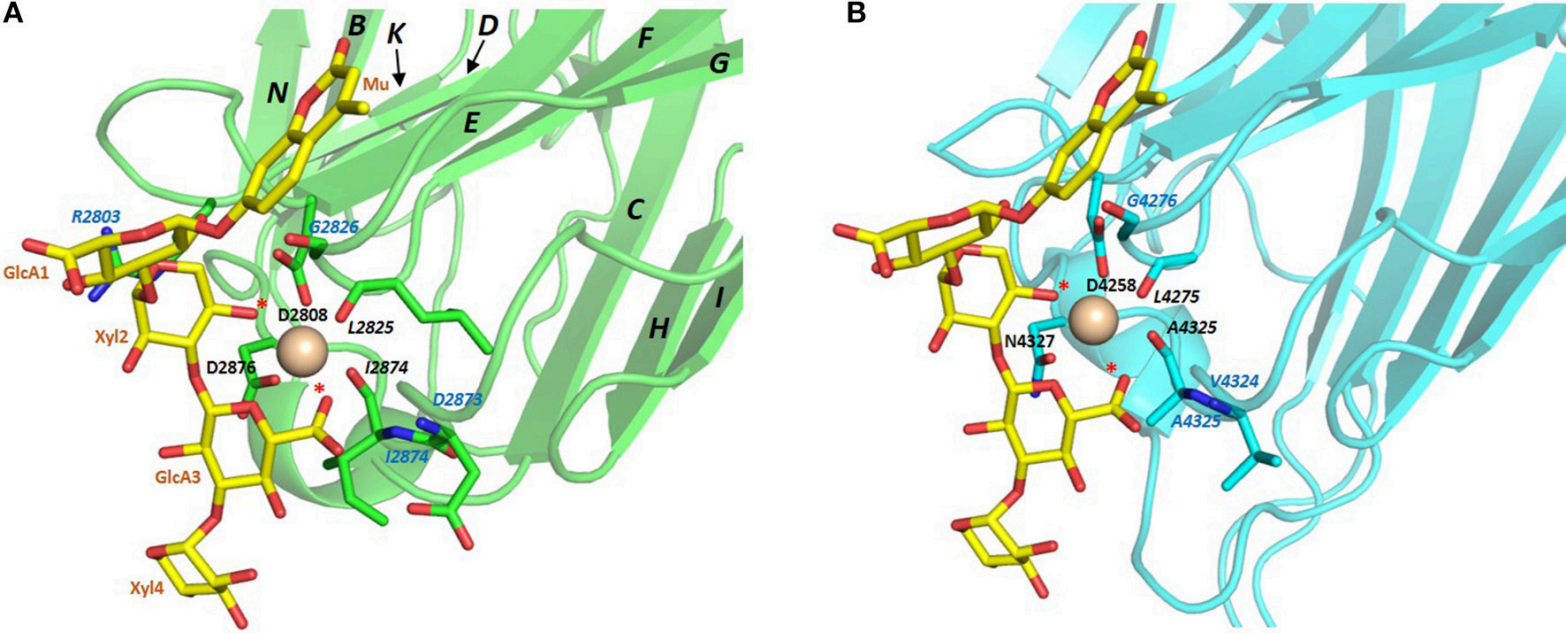
**(A)** Structure of laminin-α2 LG4 cocrystallised with a LARGE polysaccharide moiety. LG domain side chain and backbone carbonyl ligands coordinating the Ca^2+^ ion are identified with black bold and italic annotations, respectively. LG domain groups making direct interaction with the carbohydrate are identified with blue annotations; these are: R2803 side chain stacking with the Xyl2 ring; G2826 backbone carbonyl with a GlcA1 ring hydroxyl group and D2783 and I2874 backbone amide NH groups with the GlcA3 carboxyl. The two carbohydrate oxygen atoms coordinating the Ca^2+^ ion are identified with red stars (Briggs et al., 2016), PDB:5IK5. **(B)** Structure of perlecan overlaid with the disaccharide unit shown in **(A)**. To construct the model in **(B)** the perlecan LG (PDB:3SH5) and laminin-α2 LG4 domains were superimposed, and the disaccharide from laminin-α2 directly extracted onto perlecan. Apart from a small difference in the positions of the Ca^2+^ ions in the two structures, the disaccharide “fits” into the perlecan structure and provides equivalent Ca^2+^ coordinating ligands as in the laminin-α2 LG4 structure. Equivalent groups providing potential hydrogen bonds with carbohydrate (G4276; V4324; A4325) are annotated in blue. The similarities suggest that perlecan should coordinate carbohydrate units of α-DG in a manner similar to that of laminin-α2 LG4.

The conserved Ca^2+^ coordination site in LG domains of known structure comprises side chain (acidic or amide) and backbone carbonyl groups on three loops that come together on one edge of the “clam”-like LG domain, namely the loops between β-strands D-E, F-G, and J-K (see Figures 2, 3). In more detail these are (i) a highly conserved acidic side chain (usually Asp but occasionally Glu) on the D-E loop, (ii) a backbone carbonyl group on the F-G loop, and (iii) a backbone carbonyl and a side chain acidic or amide group on the long J-K loop. Water molecules likely provide two hydrogen bonds to fully coordinate the Ca^2+^ ion, and the recently resolved crystal structure of laminin-α2 LG4 in the presence of a co-crystallized α-DG polysaccharide (Briggs et al., 2016) indicates that these waters are displaced by Ca^2+^-coordinating oxygen atoms on the carbohydrate, presumably when an LG domain undergoes Ca^2+^-dependent binding. The structure reported by Briggs et al. also shows that in addition to coordinating the LG domain Ca^2+^ ion, the disaccharide moiety interacts directly with the laminin α2 LG4 domain through an interesting stacking interaction between R2803 and the GlcA1 ring, and via hydrogen bonds between backbone amide NH groups (D2873 and I2874) with the GlcA3 ring carboxyl group and between the backbone carbonyl of G2826 and GlcA1 ring hydroxyl group (Figure 3A). Although the carbohydrate-stacking arginine of laminin α2 LG4 is not conserved amongst LG domains (see Figure S1), backbone amide groups in equivalent hydrogen-bonding positions, and especially a highly conserved glycine equivalent to G2826 (see Figure 3B and Table 2) may provide common interaction points for LG domains that bind α-dystroglycan.

**Table 2:** Amino acid ligands for Ca^2+^ in X-ray structures and modeled LG domains.

**LG domain and PDB**	**D–E loop**	**F–G loop**	**J–K loop**	**Species and NCBI accession codes**
Laminin α1 LG4 2JD4 (Mg^2+^)	NQM***D***_2771_YAT	FMFD***L***_2788_GKGR	GK^a^AT***T***_2838_L***D***_2840_VERK	*Mus musculus* **P19137**
Laminin α2 LG4 5IK5 (Ca^2+^)	NHA***D***_2808_FATV	FSYD***L***_2825_GSGD	KKAD***I***_2874_L***D***_2876_VV	*Mus musculus* **Q60675**
Laminin α2 LG5 1QU0 (Ca^2+^)	QKM***D***_2982_GMG	MFHVD***N***_2999_GAG	SAST***S***_3053_A***D***_3055_TNDP	*Mus musculus* **Q60675**
Laminin α3 LG4 model on 2JD4	–	FALG***T***_3050_***D***_3051_GKKLR	LPGN***S***_3098_***T***_3099_ISIR	*Homo sapiens* **Q16787**
Laminin α5 LG3 model on 2JD4	RASP***D***_3169_GLCQ	–	PPP***E***_3233_L***Q***_3235_PQP	*Homo sapiens* **O15230**
Laminin α5 LG4 model on 2JD4	VAQ***M***_3405_***E***_3406_GLGT	–	HQGA***E***_3460_HPQP	*Homo sapiens* **O15230**
Agrin LG3 1PZ7,1PZ8 (Ca^2+^) 1PZ9 (Ca^2+^ -free)	GLERS***D***_1953_YIALA	MMYD***L***_1970_GSKP	LGAT***Q***_2020_L***D***_2022_TD	*Gallus gallus* **P31696**
Perlecan LG3 3SH5 (Ca^2+^) 3SH4 (Ca^2+^-free)	GVEVGEAGQGK***D***_4258_FISL	VFRYQ***L***_4275_GSGEAR	PGP***N***_4323_V***A***_4325_V***N***_4327_AKGS	*Homo sapiens* **P98160**
Neurexin 1α LG2 2H0B (Ca^2+^)	GKSA***D***_329_YVN	SLVIN***L***_346_GGSGAF	EDYT***M***_414_LGSDD	*Bos taurus* **XP_010808206**
Neurexin 1β LG1 2R1D (Ca^2+^)	SGLG***D***_137_YLE	VKFN***V***_154_GTDD	GRQLT***I***_236_F***N***_238_SQAT	*Rattus norvegicus* **Q63373**
Slit-1 LG1 model on 2JD4	GDN***D***_1210_HIAV	VSYD***P***_1227_GSYP	GKHY***T***_1278_LNSEA	*Homo sapiens* **O75093**
Slit-2 LG1 model on 1QU0 and 2JD4	GDK***D***_1204_HIA	ASYD***T***_1221_GSHP	NLSK***Q***_1270_S***T***_1272_L***N***_1274_F	*Homo sapiens* **O94813**
Slit-3 LG1 model on 2JD4	KGDN***D***_1209_PLA	VYD***S***_1226_***L***_1227_SSPP	QKQP***A***_1277_VGIN	*Homo sapiens* **NP_001258875**

a *K is reported as a N in the NCBI sequence: a mutation introduced to eliminate N-glycosylation. Amino acids in bold and italics provide side chain (carboxyl, amide or hydroxyl) or backbone carbonyl Ca^2+^ liganding groups in the observed or modeled Ca^2+^ binding sites. The highly conserved glycine residue that follows the Ca^2+^ liganding group in the F-G loop of many LG domains is underlined*.

## Structural Analysis and Modeling of Different α-DG Binding LG Domains

Table 1 summarizes key properties of LG domains present in proteins and proteoglycans that are known to bind α-DG and which are discussed in detail below. Particular emphasis is given to the LG-domains that bind as isolated single domains or as a tandem array. In some cases (e.g., laminin-α3-containing laminin isoforms), the possibility of α-DG binding has not been tested yet, although sequence homologies with domains having known structures allows these to be modeled and inferences made about their likely Ca^2+^ and α-DG binding.

### Laminins

**Figure 4:**
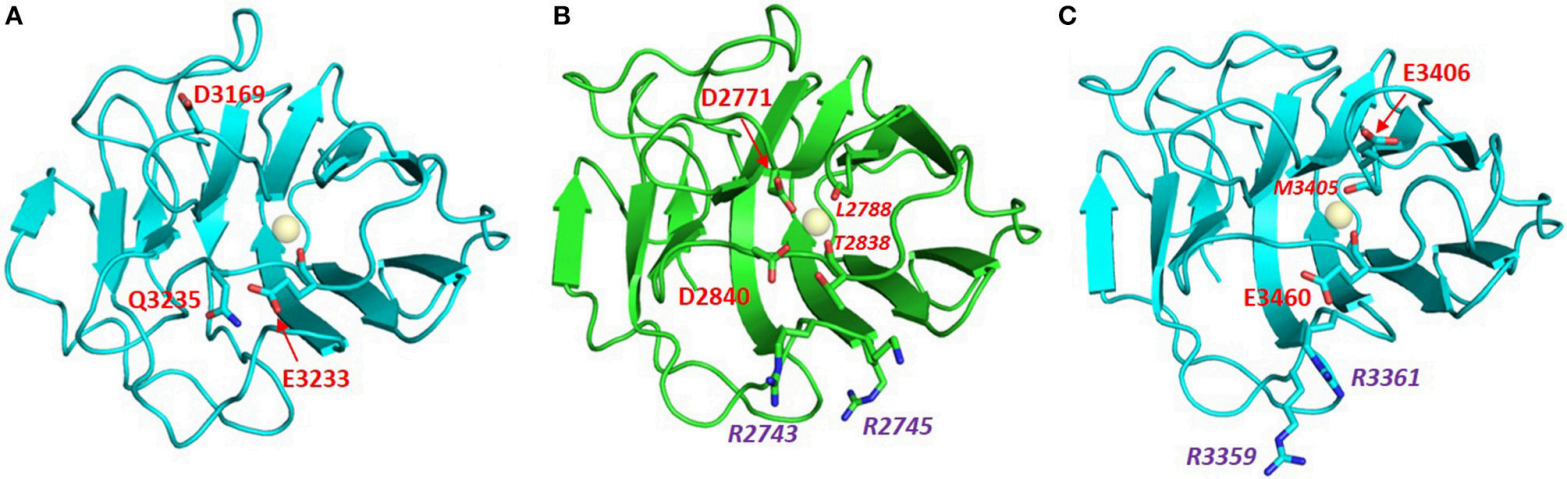
Homology models of laminin LG domains. Laminin-α5 LG3 **(A)** and laminin-α5 LG4 **(C)** built on the crystal structure template of murine laminin-α1 LG4 (**B**, PDB:2JD4, NCBI Code: P19137). Neither the LG3 nor the LG4 domain of laminin-α5 has a canonical Ca^2+^ binding site. However, each of the laminin-α5 LG domain models places potential Ca^2+^ chelating ligands near the expected position of a Ca^2+^ ion. Conformational flexibility in the loops on the edge of these domains may allow weak Ca^2+^ binding that could explain observations of EDTA attenuation of α-DG binding by laminin-α5. The laminin-α5 LG4 model has a basic patch (RHR) in a topologically-equivalent location on the B-C loop as other basic patches of other known α-DG-binding LG domains including laminin-α1 LG4 (middle). Note that 2JD4 is the murine laminin-α1 LG4 structure; the human laminin-α1 LG4 equivalent has RKK rather than RKR in this basic patch motif. In red: residues involved in Ca^2+^ binding (italic numbers denote backbone carbonyl ligands), in purple: Arg residues of the basic patch.

Laminins are hetero-trimers composed of single α, β, and γ chains. They represent the most extensively biochemically characterized family of α-DG binding partners. In fact, laminin-globular (LG) domains are named after the five domains commonly found at the C-terminal portion of all laminin α chains, typically separated by short stretches of linking amino acids (Beck et al., 1990; Timpl et al., 2000). Extensive binding analysis (Andac et al., 1999; Talts et al., 1999, 2000) as well as crystallization and X-ray structural analysis, carried out by Hohenester and colleagues (Hohenester et al., 1999; Tisi et al., 2000; Harrison et al., 2007; Carafoli et al., 2009) on several laminin LG domains expressed recombinantly, suggests the presence of a hierarchy of binding modes and affinities (see Table 1). Affinity studies have shown that the presence of tandem arrays seems to be required for efficient binding to DG (e.g., two domains as in LG4–5, or three as in LG1–3); in only a few cases can binding be supported by an isolated LG domain. Examples of the latter are LG4 of the laminin α1 chain (probably the most efficient binder) (Durbeej et al., 2001) or, with reduced affinity, LG3 of α2 or LG4 of α5 (see Figure 4).

From the viewpoint of function, the terminal LG domains seem to represent a binding *hot-spot*. For example, it has been observed that binding of LG4-5 of laminin α1 induces tyrosine phosphorylation of syntrophin, initiating a signaling pathway (Zhou et al., 2006). In a similar line of enquiry, the functional role of a specific stretch of residues spanning the loop which connects the adjacent E and F β-strands of the LG4 module and is important for syndecan and heparin binding in laminin-332 (Utani et al., 2001), has been analyzed in all the five isoforms of murine laminin α via a panel of synthetic peptides (Suzuki et al., 2003). The analysis suggested that this region is involved in several biological activities, including cell attachment and neurite outgrowth, although α-DG binding was not tested (Suzuki et al., 2003). The latter observation is consistent with the position of this loop at the opposite side of the LG domains from the Ca^2+^ and carbohydrate binding regions that are involved in α-DG binding (see Figure 2).

**Figure 5:**
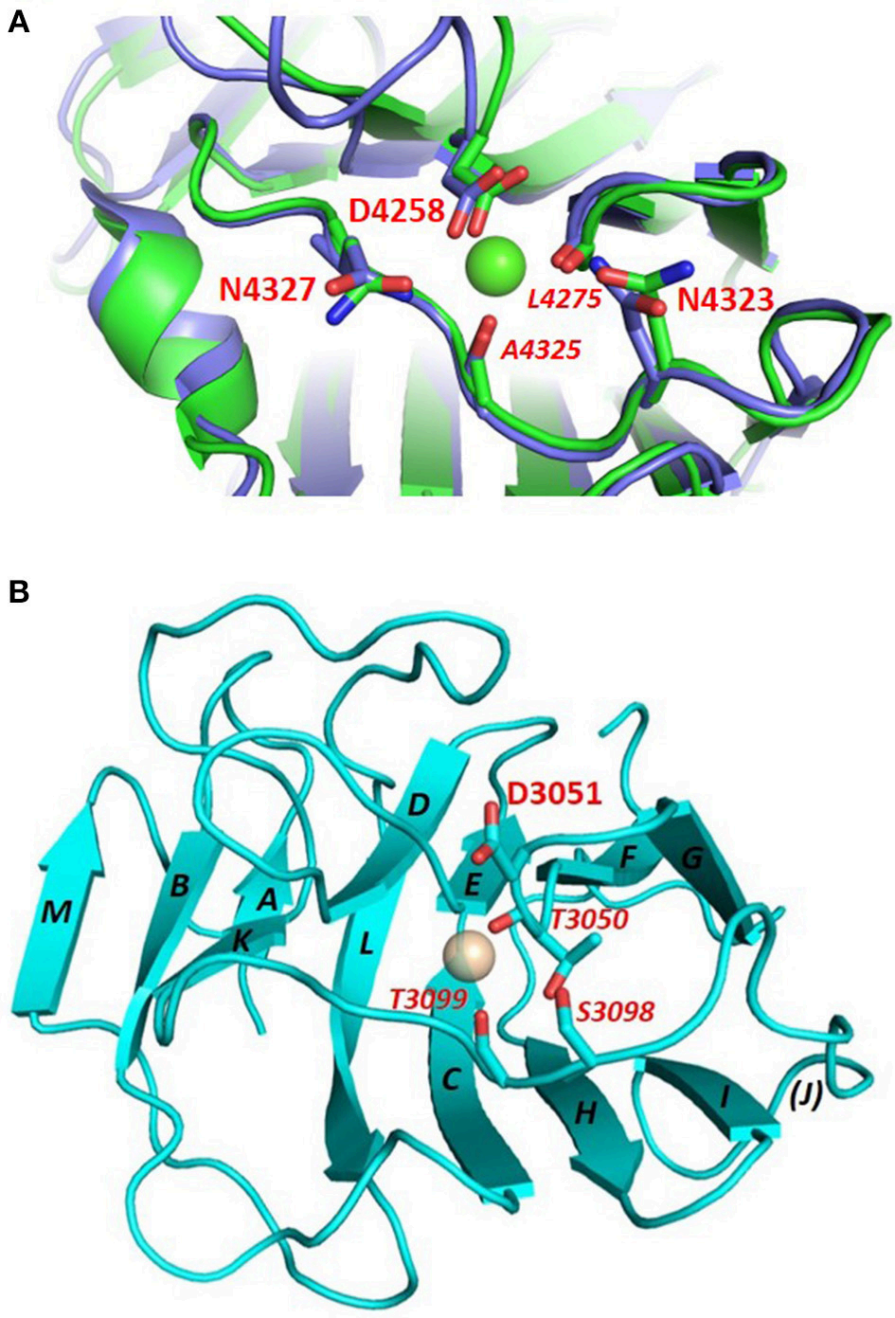
**(A)** Comparison of the Ca^2+^ binding site in Ca^2+^-bound (green) and Ca^2+^-free (blue) perlecan. Ca^2+^-bound perlecan PDB:3SH5; Ca^2+^-free perlecan PDB:3SH4. The structure of the binding site is maintained in the absence of Ca^2+^. Similar observations have been made with agrin in its free and Ca^2+^ - bound states (Stetefeld et al., 2004). The Ca^2+^ ligands are highlighted; italic type denotes backbone carbonyl ligands. **(B)** Homology model of laminin-α3 LG4. The model was built on the crystal structure template of laminin-α1 LG4 (PDB:2JD4). This domain lacks potential Ca^2+^-chelating acidic or amide groups on the D-E and J-K loops (see residues labeled in red); the side chain carboxyl (D3051) on the F-G loop is poorly positioned to chelate Ca^2+^. This domain is predicted to have a very weak Ca^2+^ binding site.

As far as calcium is concerned, it is generally accepted that this metal is required to achieve tight α-DG binding (Ervasti and Campbell, 1993; Brancaccio et al., 1995). In fact, a calcium ion must be bound to LG4, but not LG5, for efficient binding of murine laminin-α2 to α-DG (Wizemann et al., 2003), and, more recently, the importance of Ca^2+^ in LG4 of murine laminin α1 for chelating disaccharide units present on α-DG has been demonstrated (Briggs et al., 2016). Underscoring the importance of Ca^2+^ for α-DG binding, crystal structures of a number of LG domains have been solved that contain a Ca^2+^ (or Mg^2+^) ion within a structurally homologous binding site. These include LG domains of agrin, laminins, neurexins, and perlecan (Figure 5A).

The Ca^2+^ site of LG domains is a weak site with just two (laminins; agrin) or one (neurexins; perlecan; slits) negatively-charged side chains involved in chelating the metal ion (Table 2). The relatively weak binding affinity of LG domains for Ca^2+^ is consistent with a matching of Ca^2+^-binding affinity to the physiological calcium concentration in the extracellular *milieu*, which is in the low millimolar range (Hofer and Brown, 2003).

Based on sequence analysis, it has been suggested that laminin α3, α4, and α5 chains would not bind calcium (Timpl et al., 2000). It has been shown, however, that EDTA does abolish binding of α-DG to both a laminin-α5 LG domain fragment (including LG1 to LG5) expressed in bacteria and labeled with biotin (Shimizu et al., 1999), and to α5-containing laminin (Yu and Talts, 2003) or laminin-α5 and specific deletion mutants recombinantly expressed in eukaryotic cells (Ido et al., 2004). Homology models obtained here suggest that weak Ca^2+^ binding sites might be formed from non-canonical arrangements of candidate Ca^2+^ ligands (Figure 4), potentially explaining the effects of EDTA on α-DG binding to laminin-α5 LG domain fragments. Laminin-α3 is likely to represent a paradigmatic α-DG non-binder (Ferletta et al., 2003; Kikkawa et al., 2004). In fact, the nature of potential cell-surface laminin-α3 binders with roles in cell adhesions are unknown. A synthetic peptide based on the murine laminin-α3 sequence that spans the loop connecting the two adjacent E and F β-strands (within its LG4 module) does not affect cell attachment (Suzuki et al., 2003), whereas the human-based peptide does (Yokoyama et al., 2005). However, the terminal LG4-5 domains of laminin-α3 can bind syndecan 1 and 4 (Utani et al., 2001; Carulli et al., 2012). Homology modeling of laminin-α3 LG domains presented here (Figure 5B) supports the conclusion that these domains are unlikely to bind Ca^2+^. We propose that this may contribute to low α-DG affinities.

### Agrin

Neuronal agrin is a heparan sulfate proteoglycan secreted presynaptically which plays an important role in the maturation and stability of the postsynaptic element at the neuromuscular junction (NMJ) (Ruegg and Bixby, 1998). It harbors three LG domains within its C-terminal region, and analysis of isolated domains produced recombinantly showed that the first two (LG1 and LG2) are sufficient for DG binding whereas the last one, LG3 (whose structure has been solved, Stetefeld et al., 2004), lacks or shows very weak binding to α-DG yet is sufficient in isolation for the maturation of the NMJ (Gesemann et al., 1996). In addition, NMR studies have shown that the LG3 domain of agrin binds sialic acid in a Ca^2+^-dependent manner, whilst binding the glycosaminoglycans heparin and heparan sulfate bind independently of Ca^2+^. It remains unclear whether these observations may be relevant for α-dystroglycan binding to agrin (Sallum et al., 2007).

**Figure 6:**
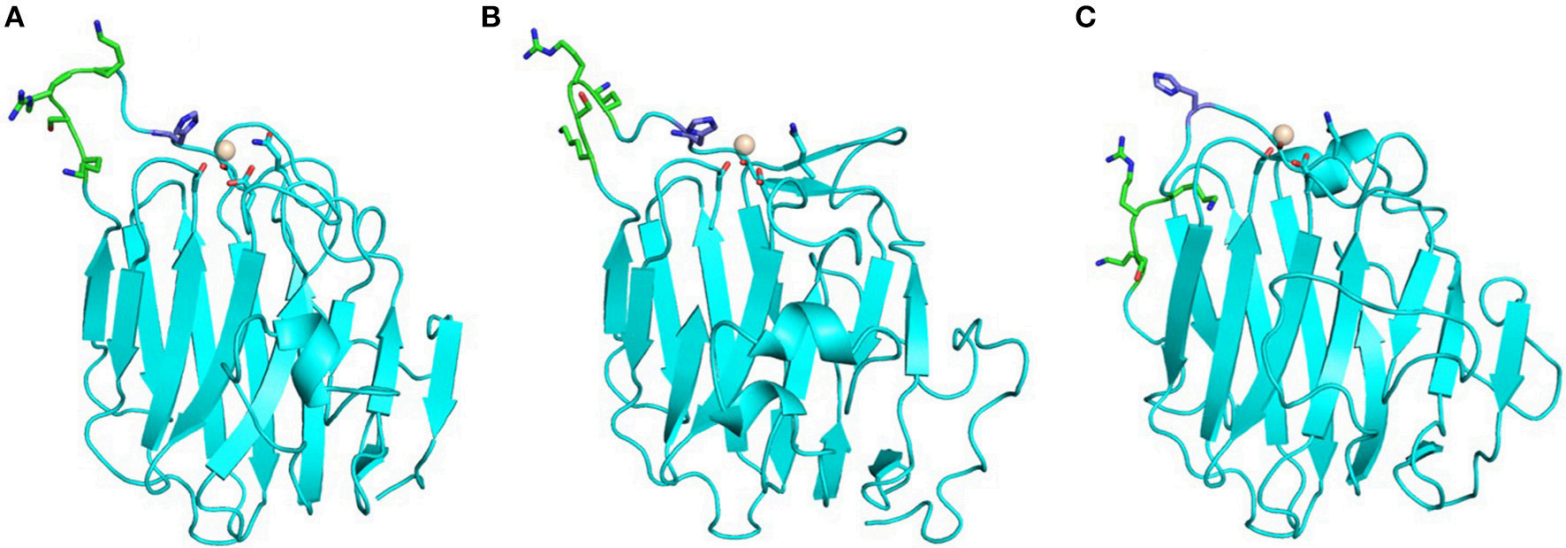
Homology models of agrin LG2 KSRK-containing splice variant. Models were obtained using as template: **(A)** human perlecan LG3 (3SH5), **(B)** chicken agrin LG3 (1PZ7) and **(C)** murine laminin-α1 LG4 (2JD4). The potential Ca^2+^ binding site is relatively well defined and independent of the structural template. The Ca^2+^ ions modeled into the putative Ca^2+^ binding sites are shown as spheres. The position of the KSRK sequence (green) that lies in the loop between the J and K strands (see text) is not well-constrained in the models. These models illustrate the variability in structures of modeled LG domains obtained when using a range of different structural templates. Note that the strongest sequence homology (≈35% identity) is between human agrin LG2 and perlecan LG3.

The LG domains of agrin are separated by EGF-like modules. Interestingly, a splice variant that includes an inserted basic stretch of residues (site A: KSRK) in the loop between the J and K strands, which introduces a heparin binding site within its LG2 domain, binds α-DG less efficiently (Campanelli et al., 1996; Gesemann et al., 1996; O'Toole et al., 1996). The homology models of agrin LG2 in Figure 6 show that the basic site A sequence (KSRK) likely lies adjacent to a putative Ca^2+^ binding site spatially homologous to the Ca^2+^ sites in LG domains of known structure, such as perlecan (see Figures 2, 6). Additional agrin splicing isoforms of different lengths (0, 8, 11, or 19 a.a.), involve a site (B) located between the last EGF-like module and the final LG3 domain. The agrin isoform A0B0 (expressed by skeletal muscle cells) shows the tightest binding to α-DG (Gesemann et al., 1996, 1998).

**Figure 7:**
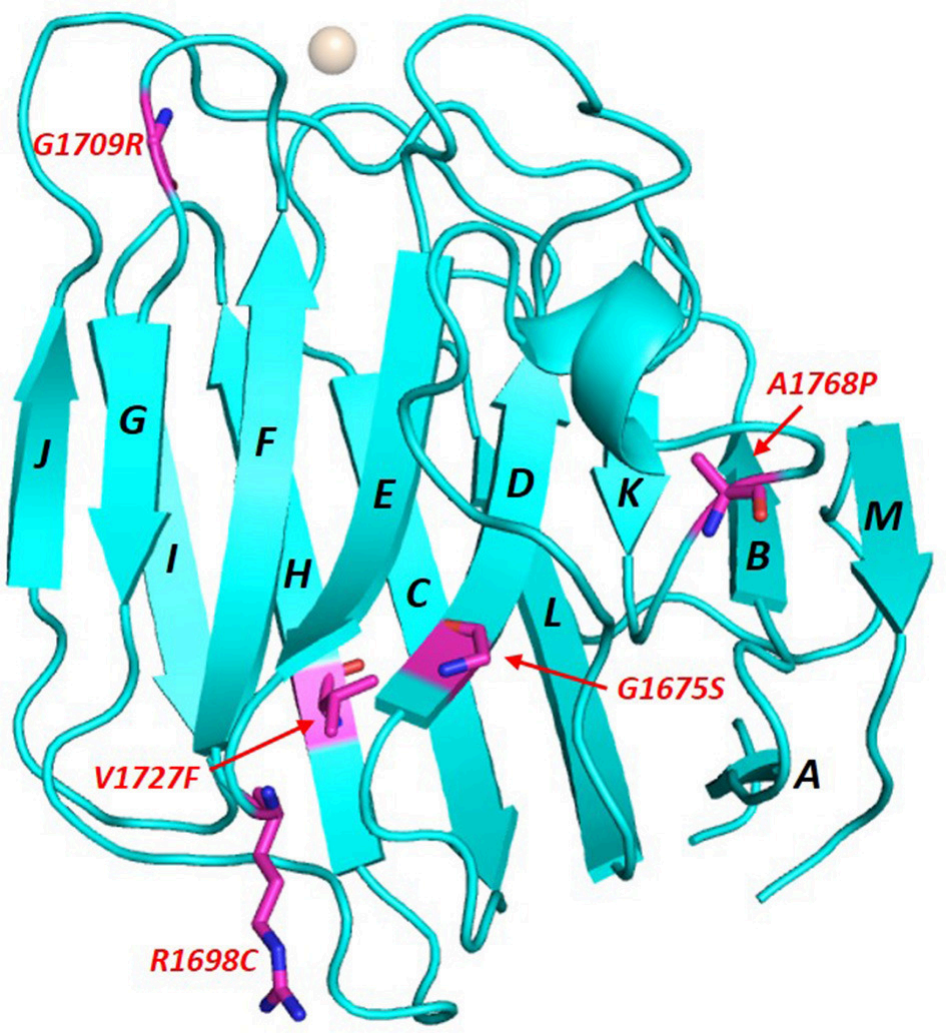
Homology model of human agrin LG2 showing the structural context of mutations that cause congenital myasthenic syndrome (CMS). The domain scaffold (strands and loops) is shown in blue with the Ca^2+^ atom in white whilst the missense mutations positions (numbering referring to human agrin, NCBI code: O00468) are reported in purple. The model was constructed using the perlecan LG3 domain (PDB: 3SH5; see Figure 2) as a template.

Several missense mutations (reported in green in Figure S1), mostly homozygous, have been identified in LG domains of agrin (Huzé et al., 2009; Maselli et al., 2012; Karakaya et al., 2017; Xi et al., 2017; Zhang et al., 2017); these are located in β-strands or inter-strand loops that may be involved in α-DG binding. The missense mutations that cause congenital myasthenic syndrome (CMS) affect the agrin LG2 domain (namely, G1675S, R1698C, G1709R, V1727F, and A1768P), which is known to be part of the α-DG binding site together with LG1 (Gesemann et al., 1996). The extent to which these mutations might affect α-DG binding and how the agrin/α-DG connection would relate to the range of CMS phenotypes have yet to be determined. For example, an instability of agrin and impaired clustering of the acetylcholine receptor (AChR) has been observed in cells containing the R1698C mutant (Xi et al., 2017), which, according to our model, is expected to lie on the E-F loop on the opposite side of the domain to the Ca^2+^ coordination site. The neighboring G1709R that lies on the FG loop near the Ca^2+^ site was not reported to affect α-DG binding (Huzé et al., 2009). The structural context of these mutations is illustrated on the model of human agrin LG2 in Figure 7.

### Perlecan

Another heparan sulfate proteoglycan which binds α-DG tightly (Friedrich et al., 1999; i.e., with Kd within the low nanomolar range, Talts et al., 1999) is perlecan. The C-terminal portion of perlecan is termed domain V and includes three LG domains and several EGF-like domains organized in the following order: LG1-EG1-EG2-LG2-EG3-EG4-LG3. The recombinant domain V, which includes all the LG domains (LG1-3), shows the tightest binding to α-DG, but LG1-2 and LG2-3 pairs can also bind; conversely, isolated perlecan LG domains did not bind in the range of ligand concentrations explored (i.e., up to 500 nM) (Friedrich et al., 1999). Perlecan domain V, renamed “endorepellin” due to its angiostatic activity (Gonzalez et al., 2005), has been involved in multiple biological functions related to its ability to modulate cytoskeletal dynamics in a calcium-dependent manner (Le et al., 2011). The high-resolution crystal structure of human perlecan LG3 has been solved (see Figures 2, 4A) and the presence of a Ca^2+^ ion confirmed, highlighting some structural similarities with other LG domains (namely, LG3 of agrin, LG2 of neurexin1α, LG5 of laminin-α2 and LG1 of neurexin1β) (Le et al., 2011). A superposition of the perlecan LG domain structure with the structure of laminin-α2 LG4 that contains a co-crystallized polysaccharide unit (Briggs et al., 2016) indicates that the α-DG binding edge of the perlecan LG domain is likely to bind disaccharide units in a similar fashion to that of laminin LG4 (Figure 3).

### Pikachurin

Pikachurin is a retinal ECM protein localized to the synaptic cleft in the photoreceptor ribbon synapse of the central nervous system, which binds α-DG in a Ca^2+^-dependent fashion (Sato et al., 2008). Within its C-terminal portion, pikachurin harbors three LG domains separated by single EGF-like domains. Binding experiments on recombinantly produced isolated domains have shown that LG2-3 are sufficient for binding with an affinity similar to LG1-3 (Kanagawa et al., 2010). This implicates pikachurin LG1 as another LG domain that could be included within the group of “non-binders” (see below). LG3 alone is unable to induce DG clustering (Omori et al., 2012), indicating that a tandem array (doublet) of LG domains is required for binding.

### Neurexins

Neurexins are neuron-specific cell surface proteins expressed at the presynaptic terminal, that bind neuronal α-DG (Sugita et al., 2001; Südhof, 2008). For an overview of the role of neurexins in synaptic organization the reader is referred to a recent review (Rudenko, 2017). In humans, there are three paralogous genes coding for neurexins 1, 2, and 3. Most of the work concerning the neurexin-DG axis has been carried out on neurexin1. Typically, the gene has two promoters: α (upstream) and β (downstream), encoding longer neurexins-α and shorter neurexins-β, each harboring LG domains within their extracellular portions. In neurexin-1, the LG domains of neurexin-α are organized with interspersed EGF-like domains as follows: LG1-EGF1-LG2-LG3-EGF2-LG4-LG5-EGF3-LG6 (see Figure 1B). Neurexin-β features only one LG domain. Interestingly, an extensive analysis of recombinant isolated domains showed that only LG2 and LG6 of neurexin1α and LG1 of neurexin1β (identical to LG6 of neurexins-α) could bind α-DG tightly and, most relevantly, in an isolated fashion. There is a plethora of neurexin splicing isoforms (Missler et al., 1998), and the forms with no inserts appear to bind better to α-DG (Sugita et al., 2001). A large body of structural work on neurexin-1 has been carried out by Rudenko and coworkers (Rudenko et al., 1999; Sheckler et al., 2006; Shen et al., 2008; Chen et al., 2011), showing that their LG domains conform to the typical jelly-roll fold motif and revealing unexpected structural similarity to legume lectins and human galectins (Rudenko et al., 1999). In fact, it has been suggested that neurexins might bind carbohydrates, and that this could have a role in the interaction with DG. In addition, neurexins bind tightly to other protein partners such as α-latrotoxin and neuroligin, and alternative splicing also modulates such binding activities (Rudenko et al., 1999). It has been shown that LG2 of neurexin1α binds calcium with K_d_ ≈ 400 μM, and that splicing may influence the affinity (Sheckler et al., 2006). Similarly, splicing was also shown to modulate the affinity for calcium of the LG domain of neurexin1β (Shen et al., 2008). However, it remains unclear how this effect may relate to α-DG binding. Interestingly, binding of α-DG and neurexophilin-1 to neurexin1α are mutually exclusive, suggesting overlapping binding epitopes, and that the binding of α-DG to neurexin can also influence the formation of trans-synaptic neurexin-neuroligin complexes (Reissner et al., 2014).

### Slits

Slit-2 is another neuronal protein that acts as a secreted axonal cue guidance factor and is reported to bind α-DG (Wright et al., 2012). Slit-2 is the only binding partner of α-DG that has a single LG domain (the other one, neurexin1β, being a cell surface receptor; see above). Although the binding constant has not been determined, it would be predicted to bind α-DG tightly. It has been shown that a pair of basic residues (in the sequence KVR) is important for DG binding, as is the presence of a coordinated calcium ion (Wright et al., 2012). There are two additional highly homologous slit paralogs, known as slit-1 (in which the KVR is not conserved) and slit-3 (in which it is), however there are no reports on their potential α-DG binding properties as yet.

**Figure 8:**
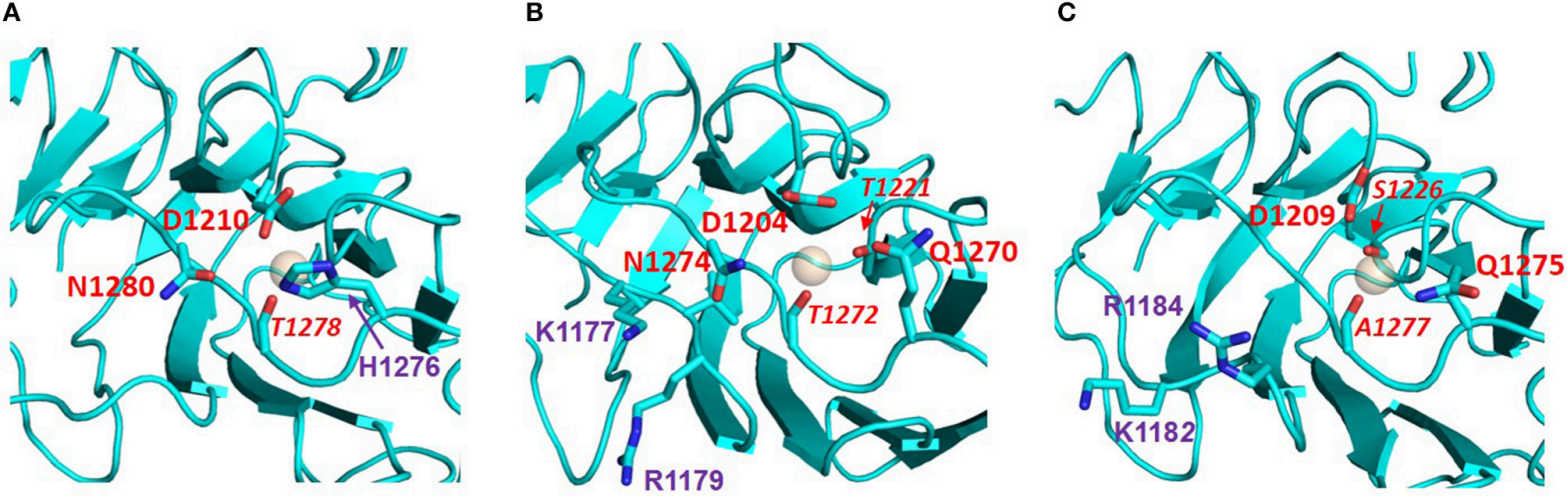
Potential Ca^2+^ sites of the LG domains of the three Slits: **(A)** Slit-1; **(B)** Slit-2; **(C)** Slit-3. Models were built on templates of LG domains with known structures (see main text) and viewed from the top edge of the β-sandwich (where “top” is equivalent to the upper part of the LG domain structures as represented in Figure 2). Slit-2 has a similar arrangement of potential Ca^2+^-chelating groups (red), as perlecan (see Figure 2). In isoforms 2 and 3 the KVR sequence (basic residues in purple) that supports α-DG binding is adjacent to the Ca^2+^ site; for comparison, the basic KSRK sequence that hinders α-DG binding in the agrin LG2 splice variant (see Figure 6) would lie to the right of the Ca^2+^ binding site in this view.

Homology models of the LG domains of the slits (Figure 8) support the presence of a canonical Ca^2+^ site in slit-2, with an arrangement of potential Ca^2+^-chelating groups that is similar to that of the structurally-defined Ca^2+^ site of perlecan (see Figure 2, with the model of slit-2 on the right for comparison); the latter binds Ca^2+^ with a K_d_ ≈ 100 μM (Le et al., 2011).

The KVR sequence required for tight binding of α-DG in slit-2 lies on the same edge of the domain as the Ca^2+^ site (Figure 8) and likely occupies a structurally equivalent position on the B-C strand loop as the corresponding RKK sequence of human laminin-α1 LG4 (RKR in murine laminin-α1 LG4; Figure 4) and the RHR sequence of laminin-α5 LG4 (Figure 4). We speculate that this basic “patch” may serve as an additional site for the recognition of another disaccharide unit, thus strengthening the interaction between α-DG and these LG domains including slit-2 (see below); the stacking interaction between R2803 and the carbohydrate Xyl2 sugar ring in the laminin-a2 LG4 structure co-crystallized with a LARGE polysaccharide unit (Briggs et al., 2016) provides a possible context for this type of interaction (see Figure 3).

The proposed structural basis for binding of slit-2 to α-DG is additionally supported by the observation that the Ca^2+^-binding edge of the domain can be modeled to interact with a disaccharide moiety in a manner similar to that identified in the structure of laminin-α2 LG4 co-crystallized with a polysaccharide moiety (Briggs et al., 2016) (i.e., very similar to the structure of perlecan LG overlaid with the disaccharide shown in Figure 3). Comparison of the equivalent LG models of slit-1 and slit-3 (Figure 8) indicates (a) that slit-3 is predicted to have a “weaker” binding site for Ca^2+^, although it has a KVR sequence in the same location as that of slit-2, and (b) that slit-1 lacks the KVR sequence and thus may have a weaker interaction with DG compared to slit-2. In addition, slit-3 lacks the highly conserved glycine residue (G1222 in slit-2; G2826 in murine laminin α2 LG4; G4276 in perlecan, see Figure 3; this Gly residue is underlined in Table 2) that may be important for maintaining structure near the Ca^2+^ binding site.

## A Visualization Code for Binding Affinities of LG Domains Toward α-DG

**Figure 9:**
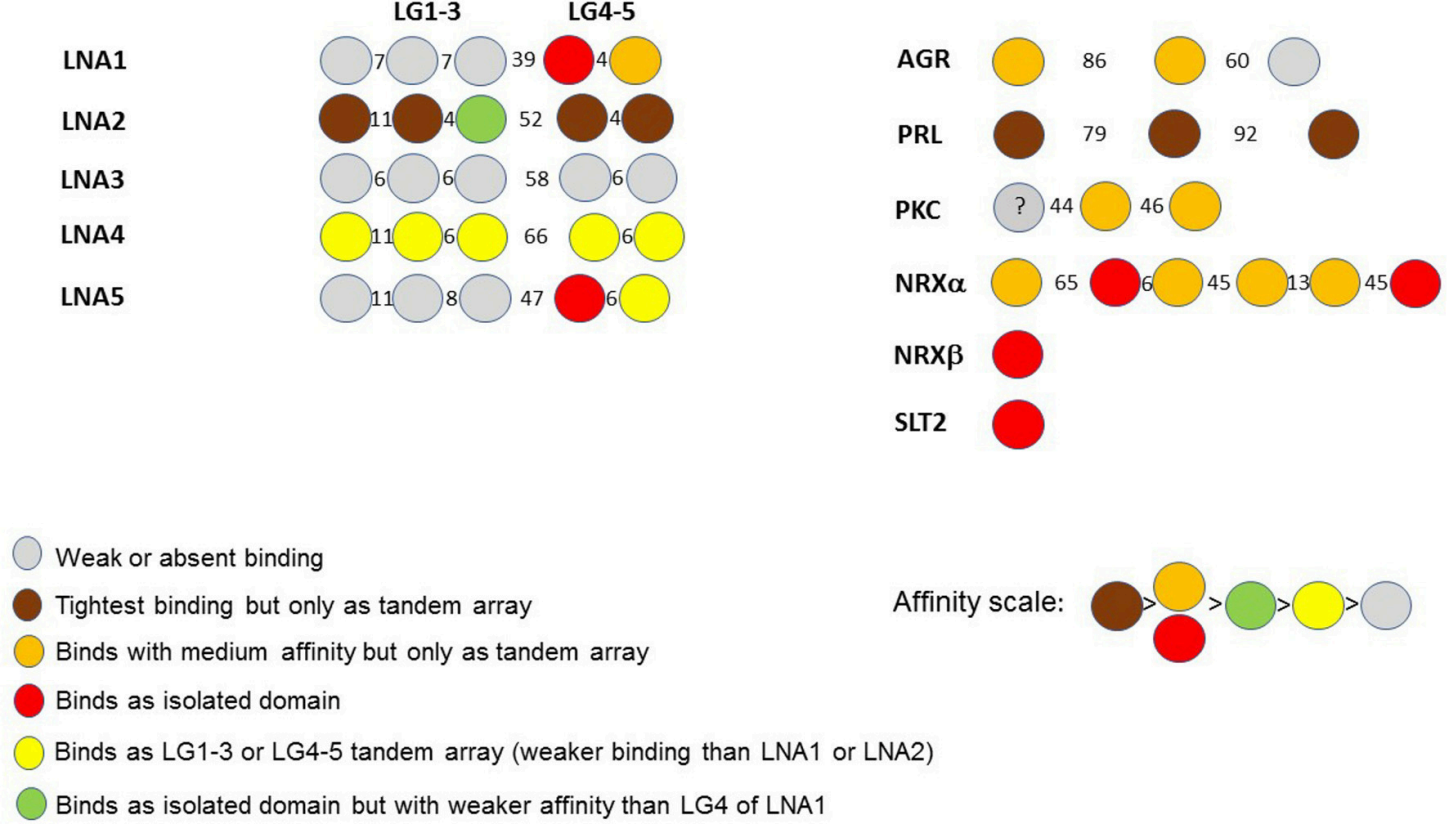
A color code for LG domain-α-dystroglycan binding affinities. Based on available biochemical information, the α-DG binding partners are classified by the binding affinities of their multiple LG domains, represented using the color code reported in the figure. The affinity scale has been estimated semi-quantitatively based on available binding data (see Sciandra et al., 2013): tighter affinity refers to the lower nanomolar range (0.1–1 nM), medium affinity to the higher nanomolar range (10–100 nM), weak or very weak binding within the micromolar range. The laminin (LN) sub-family (referring to the five different α chains, A1 to A5) is on the left whilst agrin (AGR), perlecan (PRL) and other binding partners (PKC: pikachurin, NRX: neurexin, SLT2: Slit-2) are on the right. The drawings are not to scale. Numbers between domains indicate the number of amino acids in the linkers separating them.

The binding affinities of recombinantly expressed LG domains from several DG binding partners have classically been measured or estimated by semi-quantitative solid-phase binding assays or by more quantitative Surface Plasmon Resonance. In some cases, very high apparent affinities (K_d_'s within the range 0.1–1 nM) have been reported (for a comprehensive review, see Sciandra et al., 2013). Data from these studies have allowed us to construct a scheme to characterize α-DG binding of LG domains according to the affinity and requirements for multiple LG domains (Figure 9).

It is evident that a tandem array of 2 or 3 LG domains in a row is often required for strong α-DG binding (brown in Figure 9), and these combinations (a doublet in laminin-α2; a triplet in perlecan) constitute the tightest binding detected to date. Orange and red both indicate a reduced but still significant affinity. Importantly, the few domains that apparently bind α-DG in an isolated fashion do so with relatively high affinity (see also below). However, these are not universal properties: LG3 of laminin-α2 can bind but with a weaker affinity, and tandem combinations of laminin-α4 (LG1-3 and LG4-5) and laminin-α5 (LG4-5) show a weaker binding than the tandem LGs noted above. Amongst the laminins, laminin-α3 LG domains stand out as having negligible affinity for α-DG.

Overall, it appears that the LG assembly that most favors α-DG binding is one formed by at least two sequential LG domains. Such an assembly can be found in the LG4-5 of laminins α1, α2, α4, and α5, in agrin (LG1-2) and pikachurin (LG2-3); tight or moderate binding by a three LG-domains array is found in laminin-α2 and α4 as well as in perlecan (LG1-3). Overall, the tandem arrays found in laminin-α2 (skeletal muscle isoform) and perlecan (expressed at the neuromuscular junction, NMJ) show the tightest binding to dystroglycan. This is in accordance with the biological importance of the DG adhesion complex for the stability of adult skeletal muscle and of the post-synaptic element within the peripheral nervous system (i.e., at the NMJ). Only in a limited number of cases (reported in red in Figure 9) does an isolated LG bind strongly. These include LG4 of laminin-α1, LG4 of laminin-α5, LG2, and LG6 of neurexin1α, LG1 of neurexin1β (which has an identical sequence to LG6 of neurexin1α) and LG1 of slit2.

## Are There Universal Structural Features in LG Domains That Predict Their Propensity to Bind α-Dystroglycan?

### Ca^2+^ Coordination

As described above, the typical LG module comprises a compact sandwich in which 2 β-sheets (formed by 7 β-strands each) pack against each other, and all LG domains of known structure contain a conserved Ca^2+^ coordination site. Since LG domains function in the relatively high Ca^2+^ concentration of the extracellular matrix, their Ca^2+^ binding affinity is very weak in comparison with intracellular proteins that chelate Ca^2+^ strongly such as calmodulin (4 acidic Ca^2+^-coordinating side chains, with K_d_'s within the range of 1–0.01 μM, Linse et al., 1991), and is likely to be more similar to that of the sarcoplasmic reticulum Ca^2+^ binder calsequestrin, for example, whose Ca^2+^**-**binding sites contain only one or two acidic groups and whose K_d_ is in the millimolar range (Sanchez et al., 2012).

In the two examples of LG domains for which structures with and without Ca^2+^ are known (agrin and perlecan), the Ca^2+^ binding site is essentially pre-formed (Figure 5A), indicating that Ca^2+^ binding is not required for the overall fold of the LG domain. This contrasts with very tight Ca^2+^ binders involved in Ca^2+^-induced allosteric regulation, such as calmodulin, in which structure is induced in a disordered Ca^2+^-free binding site when Ca^2+^ binds. However, NMR studies on perlecan LG domains indicate that Ca^2+^ binding, although not stringently required for correct folding, *stabilizes* the LG domain structure (Le et al., 2011). Although the Ca^2+^ site in all LG domains is at best a weak binding site and includes only one or two acidic side chain calcium-chelating ligands (see Table 2), LG domains known to lack α-DG binding activity may be especially poor Ca^2+^ binders. Homology models of the laminin-α3 LG domains, for example, indicate that the groups putatively responsible for Ca^2+^ coordination, located at the edge of the LG domain constitute a far from ideal set of potential Ca^2+^-chelators (Figure 5B).

### Linear α-DG-Binding Module(s)

**Figure 10:**
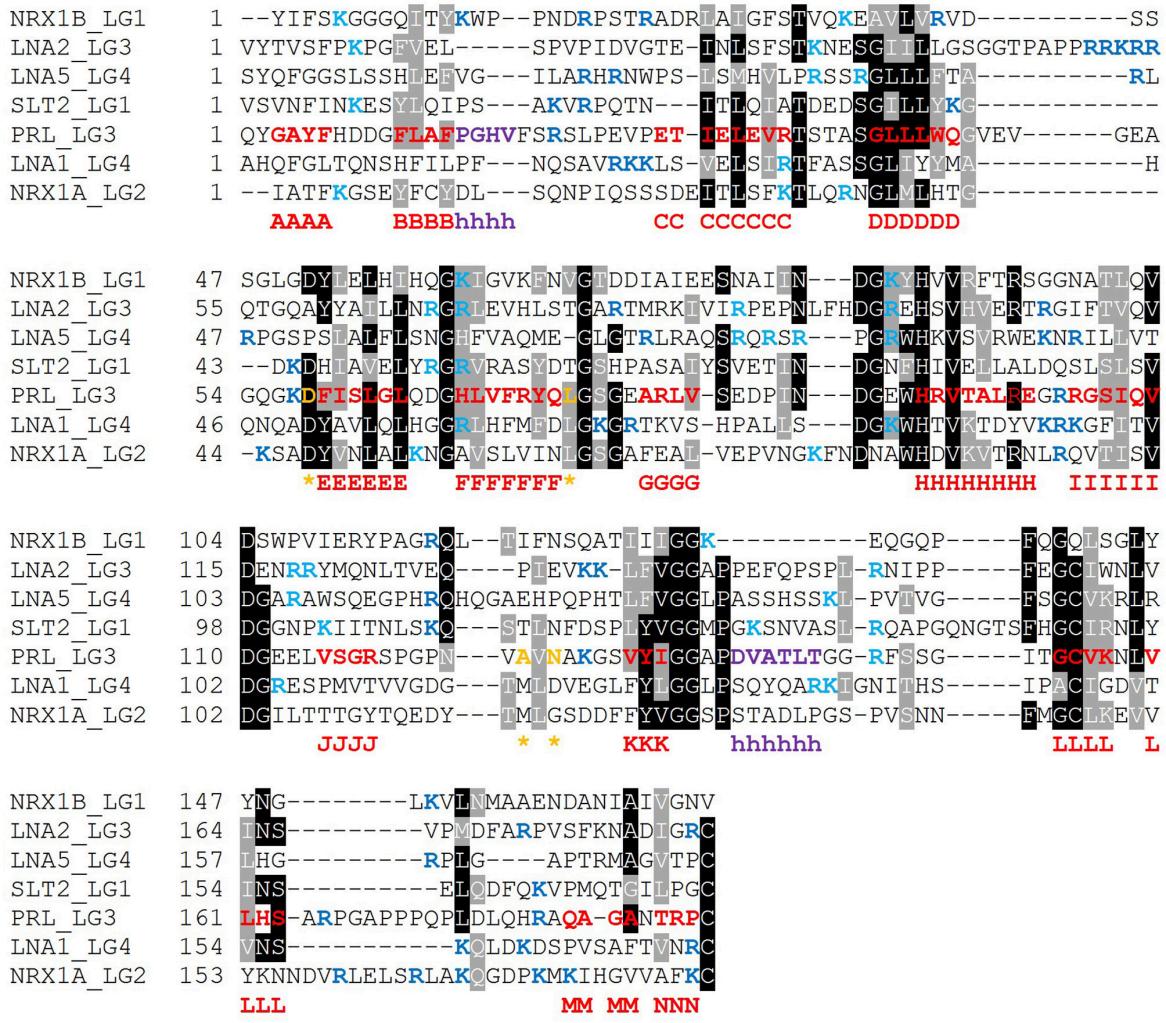
Multiple sequence alignment of selected isolated LG domains that are binders of α-DG. The sequences refer to all the strong binders identified and include also the LG3 module of the laminin α2 chain (reported to be a weaker binder). Secondary structure elements, as retrieved from the available 3D structure of human perlecan (3SH4/3SH5), are rendered as follows: β-strands, from A to N (red), α-helices (purple), calcium-coordinating amino acid positions (orange and indicated by an asterisk). Sequences were aligned in MUSCLE 3.8 and the alignment presented in Boxshade. Basic residues belonging to the loops neighboring the coordinated Ca^2+^ are reported in blue, the ones belonging to the opposite side of the domain are turquoise. The specific LG domain sequences in the alignment are identified as follows: NRX1A/1B: neurexin α1/β1, LNA1/2/5: laminin α1/α2/α5, SLT2: slit-2, PRL: perlecan.

**Figure 11:**
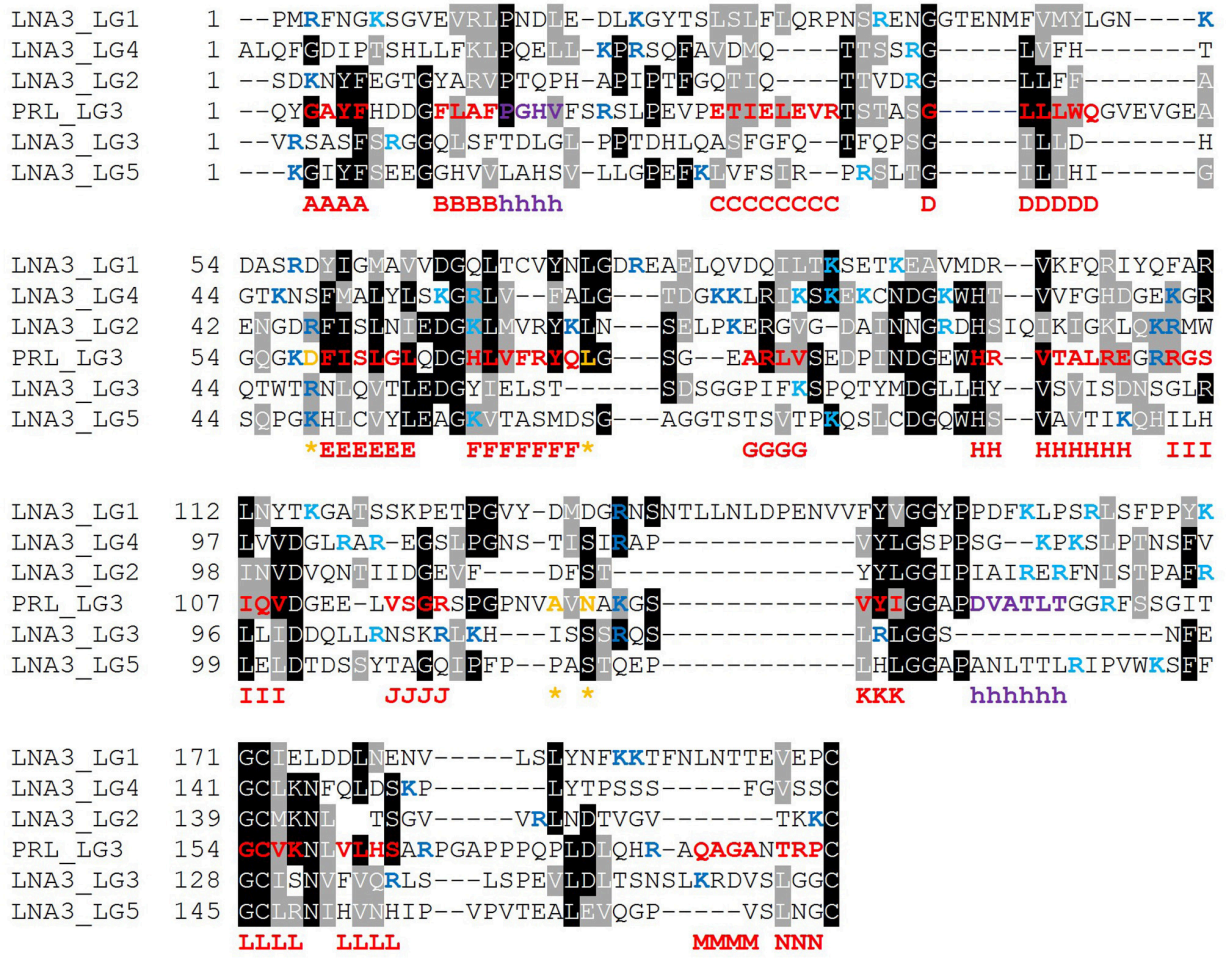
Multiple sequence alignment of selected isolated LG domains that are non-binders of α-DG. Laminin α3 has been reported not to bind α-DG. Therefore, it is assumed that none of its tandem LG domains would be able to bind α-DG. Secondary structure elements as retrieved from the available 3D structure of human perlecan (3SH4/3SH5): β-strands, from A to N (red), α-helices (purple), calcium-coordinating amino acid positions (orange and indicated by an asterisk). Basic residues belonging to the loops neighboring the coordinated Ca^2+^ are reported in blue whereas the one belonging to the opposite side of the domain in turquoise. Code: LNA3_LG1 to LNA3_LG5 (1st to 5th module from laminin α3).

It is expected that those LG domains able to bind α-DG as single domains may display some relevant sequence features that would be necessary for α-DG binding, and likewise, that useful information might be retrieved by comparative analysis of those domains which are reported not to bind α-DG. For this reason, we performed a series of alignments of selected protein sequences, with reference to human sequences. The alignment of so-called “isolated binders” is reported in Figure 10, and the LG domains of laminin-α3, which does not bind α-DG, are examined in Figure 11. An alignment of all the LG sequences under consideration was also made (Figure S1). Although perlecan domains have not been reported to bind α-DG in isolation (Friedrich et al., 1999; Talts et al., 1999), the sequence of human perlecan LG3 domain has also been included in the alignments in order to compare all the human sequences within a typical LG-domain secondary structure framework.

Based on the alignment in Figure 10, no universal short linear DG-binding motif(s) can be identified, and it is likely that a combination of sequence and structural features is required. The laminin α2 LG4 LARGE carbohydrate crystal structure (Briggs et al., 2016) shows that hydrogen bond interactions between the LG domain and carbohydrate sugar rings involve backbone amide groups (Figure 3) and these potentially important interactions may be rather relaxed with respect to amino acid sequence requirements and therefore not apparent in searches for sequence homologies. It is notable that the J strand is less conserved in the binders (Figure 10) compared to non-binders (Figure 11) and, overall, the J strand appears to be the least conserved within the entire LG domain β-strand-scaffold. In contrast to other strands, none of the residues of the J strand are highlighted in black or gray, indicating a high degree of amino acid variability (see Figure S1). This variability could play a functional role, for example in modulating α-DG recognition.

### Recurring Basic Residues Within Specific Structural Elements

The presence of scattered patches of basic residues in and around the Ca^2+^ cleft of LG domains has been suggested to represent an important requirement for α-DG binding (Harrison et al., 2007). Mutation to alanine of the two basic residues within the KVR sequence on the B-C loop of slit-2 (Wright et al., 2012) and the topologically-equivalent RKR (to AKA) in laminin-α1 LG4 (Harrison et al., 2007) abolishes α-DG binding. These observations are consistent with the expectation that basic residues facilitate the binding of LG domains to the negatively charged carbohydrate groups on α-DG. Although there were some notable discrepancies between the data collected by Harrison et al. (2007) and earlier mutational analysis from Andac et al. (1999), that might be ascribed to differences in the experimental conditions used and/or source of α-dystroglycan samples (see Harrison et al., 2007), the emerging scenario indicates that the basic residues whose mutation most perturbs α-DG binding fall on the edge of the domain that contains the Ca^2+^-binding site (Figure 2), as expected for a role in binding to carbohydrate groups on α-DG. This observation, however, is not easily generalized to the whole family of LG domains which, whether binders or non-binders, have little overall net charge at physiological pH (see Table S1).

**Figure 12:**
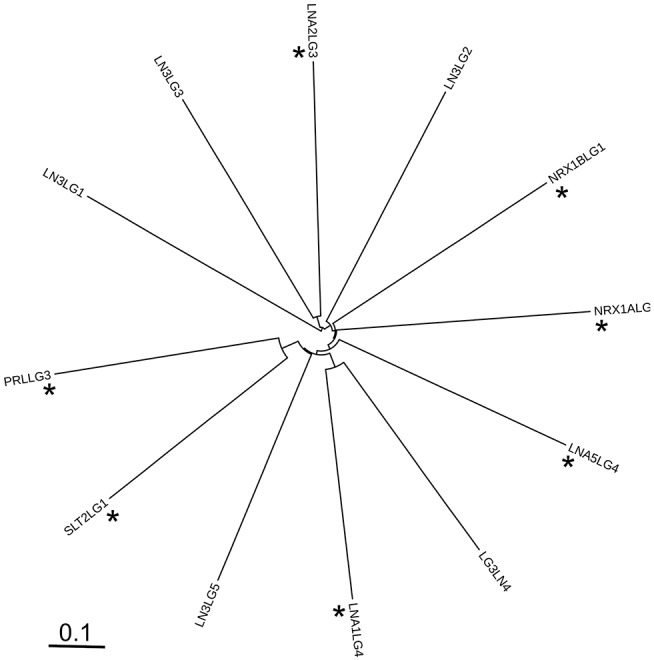
Unrooted phylogenetic diagram of the protein sequence relationships of the LG domains analyzed in this study. The alignment of 246 positions was prepared in MAFFT and the Newick output rendered in iTOL. Codenames as in Table 1. Asterisks indicate known α-DG-binding LG domains.

The sequence relationships of LG domains were also investigated by molecular phylogeny. The phylogenetic diagram resulting from the multiple sequence alignment showed the LN3-LG1 domain to be distinct from the other sequences analyzed, forming a separate branch in the tree. Within the other clades and sub-clades, there was no discrete segregation of known α-DG-binding LG domains from non-binders (Figure 12, asterisks indicate known α-DG-binding LG domains). Although this analysis is limited to the subset of experimentally-tested LG domains, the results indicate that there are no clear sequence features that distinguish α-DG binding domains from non-binders in this set, pointing to an apparent paradox intrinsic to the very nature of LG domains.

Thus, the identified features that modulate DG binding affinity, namely the presence and “strength” of the Ca^2+^ binding site and the presence of KVK-like basic patches, despite being predictive of a possible interaction, cannot alone distinguish unequivocally between strong, weak, or non-binders of α-DG.

## Is Affinity Regulated by Modularity?

**Figure 13:**
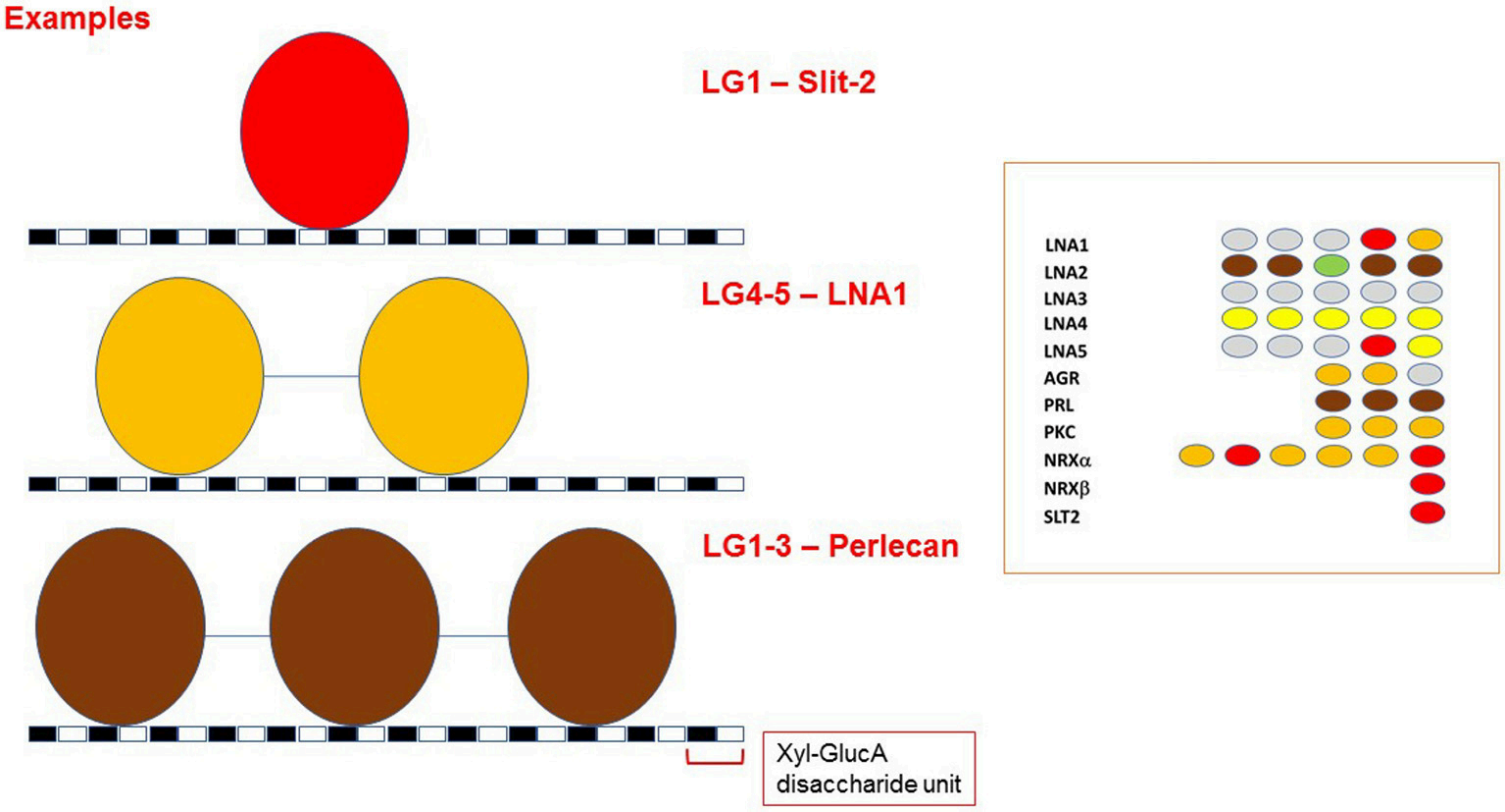
LG-domains binding modes toward α-DG. Model of how different tandem assembly combinations might be required for fine modulation of α-DG binding affinity. The distance between the two disaccharide units that interact with two adjacent LG domains has not been determined, and the cartoon only depicts an example scenario. Because of their size, tandem LG domains cannot bind to adjacent GlcA-Xyl disaccharide units. A disaccharide unit is indicated, with Xyl: Xylose and GlucA: Glucuronic acid. The grouping on the right illustrates that LG domains that are able to bind α-DG as isolated units (in red) have not been found as neighbors. For color code, see Figure 9.

Extending the analysis beyond single LG domains, tandem arrays of LG domains might constitute a way to modulate binding affinity toward α-DG through modular binding to its glycan scaffold. Indeed, the glycan polymer of α-DG has been defined as a tunable extracellular matrix protein scaffold for which increasing chain length during myogenesis enhances ligand-binding capacity (Goddeeris et al., 2013; Yoshida-Moriguchi and Campbell, 2015). Are multiple, tandem LG domains required for binding partners to recognize a series of disaccharide units on α-DG (termed matriglycan) (Willer et al., 2014)? Such a multiple binding mode could be a mechanism to increase the overall affinity between α-DG and LG domains *in vivo* (Figure 13). As a matter of fact, regulation of affinity through multivalency is a widely accepted concept in the lectin-carbohydrate field (Raman et al., 2005).

It is interesting to note that two LG domains that can act as isolated binders, (in red in inset to Figure 13), have not been found next to each other in any of the DG-binding proteins. It is tempting to propose that the assembly of repeated tight DG-binding LG domains would make the binding too strong to be compatible with normal physiology. Instead, the tandem assembly as well as the modularity of LG domains of different binding affinities could be a way to tune the interaction between α-DG and its binding partners (Figure 13). This notion could be helpful for the design of therapeutic laminin- or agrin-based molecules.

It is also unclear whether the presence of tandem arrays of LG domains may be important to favor their folding and stability or is strictly necessary to achieve a physiologically relevant affinity toward α-DG. This is an important question and further work will be required in order to address it properly.

The presence of short basic motifs (KVR or similar) in the loop between the B and C strands of LG domains could be a factor to strengthen the affinity toward α-DG in binding partners possessing only one LG domain, such as slit-2. Another protein with only one LG domain is neurexin1β, which, as a presynaptic protein, might require a relatively tight binding affinity to recognize carbohydrate moieties protruding into the synaptic cleft from the postsynaptically-located α-DG. In this regard, it should be noted that the longer neurexins-α have LG domains which are able to bind DG in an isolated fashion (LG2 and LG6). However, the KVR motif does not appear to be conserved in neurexins (Figure 10), thus other factors could be involved. The fine regulation of the affinities between DG and these neuronal proteins may make important contributions in the central nervous system for the stability of synaptic elements (Hunter et al., 2017) and/or for neuronal pathfinding (Wright et al., 2012).

The polysaccharide polymer that protrudes from α-DG represents a scaffold with multiple attachment sites for ECM binding partners. In skeletal muscle, where α-DG is extensively glycosylated, it has been suggested that this glycan scaffold would act to prevent muscular dystrophy (Goddeeris et al., 2013; Yoshida-Moriguchi and Campbell, 2015). Nevertheless, it is tempting to speculate that too tight an interaction between α-DG and laminin-α2 could also be potentially dangerous for skeletal muscle physiology as it could impair some necessary conformational plasticity (implying a dynamic behavior based on cycles of fast attachment/detachment/re-attachment) at the interface between the sarcolemma of muscle fibers and the surrounding basement membranes. The presence of a limited amount of LG domains, with a distinctively tight binding affinity for α-DG, could favor such modulation, tuning the affinity of laminin for α-DG to an optimal degree for muscle physiology.

## Future Perspective: Biomedical Significance of Differential LG Domain Affinities Toward α-Dystroglycan

The importance of studying the molecular mechanism(s) that regulate the affinity between α-DG and its binding partners is emphasized by the frequent use of laminin isoforms in gene-therapy approaches. For example, laminin-111 (containing the α1 chain) (Gawlik et al., 2010) as well as a miniaturized version of agrin (the so-called *mini-agrin*) (Moll et al., 2001), have been proposed as candidates for rescuing the severe congenital muscular dystrophy phenotypes in which laminin-211 is absent. Both of these contain LG domains known to interact strongly with α-DG. Recently, the crucial role played by the affinity of different binding partners toward α-DG has been highlighted by the finding that within the embryonic heart, agrin muscle isoform (A0B0), instead of laminin-2, is the preferential α-DG binder. During development, agrin binding to α-DG promotes the release of the transcription factor YAP (yes-associated protein 1) from sequestration by the dystrophin-glycoprotein complex (Morikawa et al., 2017), eventually allowing YAP to reach the nucleus and trigger the regeneration of cardiac myofibers (Bassat et al., 2017). Therefore, through a deeper knowledge of the molecular basis of LG domain affinity toward α-DG, strategies to modulate these interactions may be developed, and further therapeutic avenues for the treatment of severe neuromuscular disorders or for triggering regeneration procedures in the adult myocardium could be explored (Eroglu and Chien, 2017).

## Data Availability

The authors declare that all data generated for this study are included in the manuscript and the Supplementary Files.

## Author Contributions

MGB and AB conceived the project. CD performed the modeling. JA performed the tree analysis. AB carried out the sequence alignment analysis. CD, MGB, JA, and AB analyzed the data. CD and AB wrote the paper. All authors reviewed and contributed to the various draft versions of the manuscript. All authors read and approved the final manuscript.

### Conflict of Interest Statement

The authors declare that the research was conducted in the absence of any commercial or financial relationships that could be construed as a potential conflict of interest.

## References

[B1] Adams J. C. Brancaccio A. (2015). The evolution of the dystroglycan complex, a major mediator of muscle integrity. Biol. Open. 4, 1163–1179. 10.1242/bio.012468 26319583 PMC4582122

[B2] Adams J. C. Lawler J. (2011). The thrombospondins. Cold Spring Harb. Perspect. Biol. 3:a009712. 10.1101/cshperspect.a009712 21875984 PMC3179333

[B3] Andac Z. Sasaki T. Mann K. Brancaccio A. Deutzmann R. Timpl R. (1999). Analysis of heparin, α-dystroglycan and sulfatide binding to the G domain of the laminin α1 chain by site-directed mutagenesis. J. Mol. Biol. 287, 253–264. 10.1006/jmbi.1999.2606 10080889

[B4] Barresi R. Campbell K. P. (2006). Dystroglycan: from biosynthesis to pathogenesis of human disease. J. Cell Sci. 119, 199–207. 10.1242/jcs.02814 16410545

[B5] Bassat E. Mutlak Y. E. Genzelinakh A. Shadrin I. Y. Baruch Umansky K. Yifa O.. (2017). The extracellular matrix protein agrin promotes heart regeneration in mice. Nature 547, 179–184. 10.1038/nature22978 28581497 PMC5769930

[B6] Beck K. Hunter I. Engel J. (1990). Structure and function of laminin: anatomy of a multidomain glycoprotein. FASEB J. 4, 148–160. 10.1096/fasebj.4.2.2404817 2404817

[B7] Bozic D. Sciandra F. Lamba D. Brancaccio A. (2004). The structure of the N-terminal region of murine skeletal muscle α-dystroglycan discloses a modular architecture. J. Biol. Chem. 279, 44812–44816. 10.1074/jbc.C400353200 15326183

[B8] Brancaccio A. Schulthess T. Gesemann M. Engel J. (1995). Electron microscopic evidence for a mucin-like region in chick muscle α-dystroglycan. FEBS Lett. 368, 139–142. 10.1016/0014-5793(95)00628-M 7615068

[B9] Briggs D. C. Yoshida-Moriguchi T. Zheng T. Venzke D. Anderson M. E. Strazzulli A. (2016). Structural basis of laminin binding to the LARGE glycans on dystroglycan. Nat. Chem. Biol. 12, 810–814. 10.1038/nchembio.2146 27526028 PMC5030134

[B10] Campanelli J. T. Gayer G. G. Scheller R. H. (1996). Alternative RNA splicing that determines agrin activity regulates binding to heparin and α-dystroglycan. Development 122, 1663–1672. 8625852 10.1242/dev.122.5.1663

[B11] Carafoli F. Clout N. J. Hohenester E. (2009). Crystal structure of the LG1-3 region of the laminin α2 chain. J. Biol. Chem. 284, 22786–22792. 10.1074/jbc.M109.026658 19553699 PMC2755686

[B12] Carulli S. Beck K. Dayan G. Boulesteix S. Lortat-Jacob H. Rousselle P. (2012). Cell surface proteoglycans syndecan-1 and -4 bind overlapping but distinct sites in laminin α3 LG45 protein domain. J. Biol. Chem. 287, 12204–12216. 10.1074/jbc.M111.300061 22351752 PMC3320972

[B13] Chen F. Venugopal V. Murray B. Rudenko G. (2011). The structure of neurexin 1α reveals features promoting a role as synaptic organizer. Structure 19, 779–789. 10.1016/j.str.2011.03.012 21620716 PMC3134934

[B14] Durbeej M. Talts J. F. Henry M. D. Yurchenco P. D. Campbell K. P. Ekblom P. (2001). Dystroglycan binding to laminin α1LG4 module influences epithelial morphogenesis of salivary gland and lung *in vitro*. Differentiation 69, 121–134. 10.1046/j.1432-0436.2001.690206.x 11798066

[B15] Eroglu E. Chien K. R. (2017). Heart regeneration 4.0: matrix medicine. Dev. Cell. 42, 7–8. 10.1016/j.devcel.2017.06.017 28697334

[B16] Ervasti J. M. Campbell K. P. (1993). A role for the dystrophin-glycoprotein complex as a transmembrane linker between laminin and actin. J. Cell Biol. 122, 809–823. 10.1083/jcb.122.4.809 8349731 PMC2119587

[B17] Ferletta M. Kikkawa Y. Yu H. Talts J. F. Durbeej M. Sonnenberg A.. (2003). Opposing roles of integrin α6Aβ1 and dystroglycan in laminin-mediated extracellular signal-regulated kinase activation. Mol. Biol. Cell. 14, 2088–2103. 10.1091/mbc.e03-01-0852 12802077 PMC165099

[B18] Friedrich M. V. Göhring W. Mörgelin M. Brancaccio A. David G. Timpl R. (1999). Structural basis of glycosaminoglycan modification and of heterotypic interactions of perlecan domain V. J Mol Biol. 294, 259–270. 10.1006/jmbi.1999.3259 10556044

[B19] Gawlik K. I. Akerlund M. Carmignac V. Elamaa H. Durbeej M. (2010). Distinct roles for laminin globular domains in laminin α1 chain mediated rescue of murine laminin α2 chain deficiency. PLoS ONE 5:e11549. 10.1371/journal.pone.0011549 20657839 PMC2906511

[B20] Gesemann M. Brancaccio A. Schumacher B. Ruegg M. A. (1998). Agrin is a high-affinity binding protein of dystroglycan in non-muscle tissue. J. Biol. Chem. 273, 600–605. 10.1074/jbc.273.1.600 9417121

[B21] Gesemann M. Cavalli V. Denzer A. J. Brancaccio A. Schumacher B. Ruegg M. A. (1996). Alternative splicing of agrin alters its binding to heparin, dystroglycan, and the putative agrin receptor. Neuron 16, 755–767. 10.1016/S0896-6273(00)80096-3 8607994

[B22] Goddeeris M. M. Wu B. Venzke D. Yoshida-Moriguchi T. Saito F. Matsumura K. (2013). LARGE glycans on dystroglycan function as a tunable matrix scaffold to prevent dystrophy. Nature 503, 136–140. 10.1038/nature12605 24132234 PMC3891507

[B23] Gonzalez E. M. Reed C. C. Bix G. Fu J. Zhang Y. Gopalakrishnan B.. (2005). BMP-1/Tolloid-like metalloproteases process endorepellin, the angiostatic C-terminal fragment of perlecan. J. Biol. Chem. 280, 7080–7087. 10.1074/jbc.M409841200 15591058

[B24] Harrison D. Hussain S. A. Combs A. C. Ervasti J. M. Yurchenco P. D. Hohenester E. (2007). Crystal structure and cell surface anchorage sites of laminin α1LG4-5. J. Biol. Chem. 282, 11573–11581. 10.1074/jbc.M610657200 17307732 PMC2675182

[B25] Hofer A. M. Brown E. M. (2003). Extracellular calcium sensing and signalling. Nat. Rev. Mol. Cell Biol. 4, 530–538. 10.1038/nrm1154 12838336

[B26] Hohenester E. Tisi D. Talts J. F. Timpl R. (1999). The crystal structure of a laminin G-like module reveals the molecular basis of α-dystroglycan binding to laminins, perlecan, and agrin. Mol. Cell. 4, 783–792. 10.1016/S1097-2765(00)80388-3 10619025

[B27] Hopf C. Hoch W. (1996). Agrin binding to α-dystroglycan. Domains of agrin necessary to induce acetylcholine receptor clustering are overlapping but not identical to the α-dystroglycan-binding region. J. Biol. Chem. 271, 5231–5236. 8617807 10.1074/jbc.271.9.5231

[B28] Hunter D. D. Manglapus M. K. Bachay G. Claudepierre T. Dolan M. W. Gesuelli K. A.. (2017). CNS synapses are stabilized trans-synaptically by laminins and laminin-interacting proteins. J. Comp. Neurol. 527, 67–86. 10.1002/cne.24338 29023785

[B29] Huzé C. Bauché S. Richard P. Chevessier F. Goillot E. Gaudon K.. (2009). Identification of an agrin mutation that causes congenital myasthenia and affects synapse function. Am. J. Hum. Genet. 85, 155–167. 10.1016/j.ajhg.2009.06.015 19631309 PMC2725239

[B30] Hynes R. O. (2012). The evolution of metazoan extracellular matrix. J. Cell Biol. 196, 671–679. 10.1083/jcb.201109041 22431747 PMC3308698

[B31] Ido H. Harada K. Futaki S. Hayashi Y. Nishiuchi R. Natsuka Y.. (2004). Molecular dissection of the α-dystroglycan- and integrin-binding sites within the globular domain of human laminin-10. J. Biol. Chem. 279, 10946–10954. 10.1074/jbc.M313626200 14701821

[B32] Kanagawa M. Omori Y. Sato S. Kobayashi K. Miyagoe-Suzuki Y. Takeda S.. (2010). Post-translational maturation of dystroglycan is necessary for pikachurin binding and ribbon synaptic localization. J. Biol. Chem. 285, 31208–31216. 10.1074/jbc.M110.116343 20682766 PMC2951195

[B33] Kanagawa M. Toda T. (2018). Ribitol-phosphate-a newly identified posttranslational glycosylation unit in mammals: structure, modification enzymes and relationship to human diseases. J. Biochem. 163, 359–369. 10.1093/jb/mvy020 29394359

[B34] Karakaya M. Ceyhan-Birsoy O. Beggs A. H. Topaloglu H. (2017). A novel missense variant in the AGRN gene; congenital myasthenic syndrome presenting with head drop. J. Clin. Neuromuscul. Dis. 18, 147–151. 10.1097/CND.0000000000000132 28221305 PMC5436270

[B35] Kikkawa Y. Yu H. Genersch E. Sanzen N. Sekiguchi K. Fässler R.. (2004). Laminin isoforms differentially regulate adhesion, spreading, proliferation, and ERK activation of β1 integrin-null cells. Exp. Cell Res. 300, 94–108. 10.1016/j.yexcr.2004.06.031 15383318

[B36] Le B. V. Kim H. Choi J. Kim J. H. Hahn M. J. Lee C.. (2011). Crystal structure of the LG3 domain of endorepellin, an angiogenesis inhibitor. J. Mol. Biol. 414, 231–242. 10.1016/j.jmb.2011.09.048 21996443

[B37] Linse S. Helmersson A. Forsen S. (1991). Calcium binding to calmodulin and its globular domains. J. Biol. Chem. 266, 8050–8054. 1902469

[B38] Manya H. Endo T. (2017). Glycosylation with ribitol-phosphate in mammals: new insights into the O-mannosyl glycan. Biochim. Biophys. Acta Gen. Subj. 1861, 2462–2472. 10.1016/j.bbagen.2017.06.024 28711406

[B39] Maselli R. A. Fernandez J. M. Arredondo J. Navarro C. Ngo M. Beeson D.. (2012). LG2 agrin mutation causing severe congenital myasthenic syndrome mimics functional characteristics of non-neural (z-) agrin. Hum. Genet. 131, 1123–1135. 10.1007/s00439-011-1132-4 22205389 PMC4795461

[B40] McDearmon E. L. Combs A. C. Sekiguchi K. Fujiwara H. Ervasti J. M. (2006). Brain α-dystroglycan displays unique glycoepitopes and preferential binding to laminin-10/11. FEBS Lett. 580, 3381–3385. 10.1016/j.febslet.2006.05.010 16709410

[B41] Missler M. Fernandez-Chacon R. Südhof T. C. (1998). The making of neurexins. J. Neurochem. 71, 1339–1347. 10.1046/j.1471-4159.1998.71041339.x 9751164

[B42] Moll J. Barzaghi P. Lin S. Bezakova G. Lochmüller H. Engvall E.. (2001). An agrin minigene rescues dystrophic symptoms in a mouse model for congenital muscular dystrophy. Nature 413, 302–307. 10.1038/35095054 11565031

[B43] Morikawa Y. Heallen T. Leach J. Xiao Y. Martin J. F. (2017). Dystrophin-glycoprotein complex sequesters Yap to inhibit cardiomyocyte proliferation. Nature 547, 227–231. 10.1038/nature22979 28581498 PMC5528853

[B44] Omori Y. Araki F. Chaya T. Kajimura N. Irie S. Terada K.. (2012). Presynaptic dystroglycan-pikachurin complex regulates the proper synaptic connection between retinal photoreceptor and bipolar cells. J. Neurosci. 32, 6126–6137. 10.1523/JNEUROSCI.0322-12.2012 22553019 PMC6622127

[B45] O'Toole J. J. Deyst K. A. Bowe M. A. Nastuk M. A. McKechnie B. A. Fallon J. R. (1996). Alternative splicing of agrin regulates its binding to heparin α-dystroglycan, and the cell surface. Proc. Natl. Acad. Sci. U.S.A. 93, 7369–7374. 10.1073/pnas.93.14.7369 8693000 PMC38991

[B46] Özbek S. Balasubramainian P Chiquet-Ehrismann, R. Tucker R. P. Adams J. C. (2010). The evolution of extracellular matrix. Mol. Biol. Cell 21, 4300–4306. 10.1091/mbc.e10-03-0251 21160071 PMC3002383

[B47] Raman R. Raguram S. Venkataraman G. Paulson J. C. Sasisekharan R. (2005). Glycomics: an integrated systems approach to structure-function relationships of glycans. Nat Methods. 2, 817–824. 10.1038/nmeth807 16278650

[B48] Reissner C. Stahn J. Breuer D. Klose M. Pohlentz G. Mormann M.. (2014). Dystroglycan binding to α-neurexin competes with neurexophilin-1 and neuroligin in the brain. J. Biol. Chem. 289, 27585–27603. 10.1074/jbc.M114.595413 25157101 PMC4183798

[B49] Rudenko G. (2017). Dynamic control of synaptic adhesion and organizing molecules in synaptic plasticity. Neural. Plast. 2017:6526151. 10.1155/2017/6526151 28255461 PMC5307005

[B50] Rudenko G. Hohenester E. Muller Y. A. (2001). LG/LNS domains: multiple functions – one business end? Trends Biochem. Sci. 26, 363–368. 10.1016/S0968-0004(01)01832-1 11406409

[B51] Rudenko G. Nguyen T. Chelliah Y. Südhof T. C. Deisenhofer J. (1999). The structure of the ligand-binding domain of neurexin Iβ: regulation of LNS domain function by alternative splicing. Cell 99, 93–101. 10.1016/S0092-8674(00)80065-3 10520997

[B52] Ruegg M. A. Bixby J. L. (1998). Agrin orchestrates synaptic differentiation at the vertebrate neuromuscular junction. Trends Neurosci. 21, 22–27. 10.1016/S0166-2236(97)01154-5 9464682

[B53] Sallum C. O. Kammerer R. A. Alexandrescu A. T. (2007). Thermodynamic and structural studies of carbohydrate binding by the agrin-G3 domain. Biochemistry 46, 9541–9550. 10.1021/bi7006383 17649979 PMC2111043

[B54] Sanchez E. J. Lewis K. M. Danna B. R. Kang C. (2012). High-capacity Ca^2+^ binding of human skeletal calsequestrin. J. Biol. Chem. 287, 11592–11601. 10.1074/jbc.M111.335075 22337878 PMC3322862

[B55] Sato S. Omori Y. Katoh K. Kondo M. Kanagawa M. Miyata K.. (2008). Pikachurin, a dystroglycan ligand, is essential for photoreceptor ribbon synapse formation. Nat. Neurosci. 11, 923–931. 10.1038/nn.2160 18641643

[B56] Sciandra F. Bozzi M. Bigotti M. G. Brancaccio A. (2013). The multiple affinities of α-dystroglycan. Curr. Protein Pept. Sci. 14, 626–634. 10.2174/1389203711209070644 24206164

[B57] Sheckler L. R. Henry L. Sugita S. Südhof T. C. Rudenko G. (2006). Crystal structure of the second LNS/LG domain from neurexin 1α: Ca^2+^ binding and the effects of alternative splicing. J. Biol. Chem. 281, 22896–22905. 10.1074/jbc.M603464200 16772286 PMC2293330

[B58] Sheikh M. O. Halmo S. M. Wells L. (2017). Recent advancements in understanding mammalian O-mannosylation. Glycobiology 27, 806–819. 10.1093/glycob/cwx062 28810660 PMC6082599

[B59] Shen K. C. Kuczynska D. A. Wu I. J. Murray B. H. Sheckler L. R. Rudenko G. (2008). Regulation of neurexin 1β tertiary structure and ligand binding through alternative splicing. Structure 16, 422–431. 10.1016/j.str.2008.01.005 18334217 PMC2346596

[B60] Shimizu H. Hosokawa H. Ninomiya H. Miner J. H. Masaki T. (1999). Adhesion of cultured bovine aortic endothelial cells to laminin-1 mediated by dystroglycan. J. Biol. Chem. 274, 11995–12000. 10.1074/jbc.274.17.11995 10207021

[B61] Stetefeld J. Alexandrescu A. T. Maciejewski M. W. Jenny M. Rathgeb-Szabo K. Schulthess T.. (2004). Modulation of agrin function by alternative splicing and Ca^2+^ binding. Structure 12, 503–515. 10.1016/j.str.2004.02.001 15016366

[B62] Südhof T. C. (2008). Neuroligins and neurexins link synaptic function to cognitive disease. Nature 455, 903–911. 10.1038/nature07456 18923512 PMC2673233

[B63] Sugita S. Saito F. Tang J. Satz J. Campbell K. Südhof T. C. (2001). A stoichiometric complex of neurexins and dystroglycan in brain. J. Cell Biol. 154, 435–445. 10.1083/jcb.200105003 11470830 PMC2150755

[B64] Suzuki N. Nakatsuka H. Mochizuki M. Nishi N. Kadoya Y. Utani A.. (2003). Biological activities of homologous loop regions in the laminin alpha chain G domains. J. Biol. Chem. 278, 45697–45705. 10.1074/jbc.M304667200 12933811

[B65] Talts J. F. Andac Z. Göhring W. Brancaccio A. Timpl R. (1999). Binding of the G domains of laminin α1 and α2 chains and perlecan to heparin, sulfatides, α-dystroglycan and several extracellular matrix proteins. EMBO J. 18, 863–870. 10.1093/emboj/18.4.863 10022829 PMC1171179

[B66] Talts J. F. Mann K. Yamada Y. Timpl R. (1998). Structural analysis and proteolytic processing of recombinant G domain of mouse laminin α2 chain. FEBS Lett. 426, 71–76. 10.1016/S0014-5793(98)00312-3 9598981

[B67] Talts J. F. Sasaki T. Miosge N. Göhring W. Mann K. Mayne R.. (2000). Structural and functional analysis of the recombinant G domain of the laminin α4 chain and its proteolytic processing in tissues. J. Biol. Chem. 275, 35192–35199. 10.1074/jbc.M003261200 10934193

[B68] Talts J. F. Timpl R. (1999). Mutation of a basic sequence in the laminin α2LG3 module leads to a lack of proteolytic processing and has different effects on β1 integrin-mediated cell adhesion and α-dystroglycan binding. FEBS Lett. 1999, 319–323. 10.1016/S0014-5793(99)01180-1 10570932

[B69] Timpl R. Tisi D. Talts J. F. Andac Z. Sasaki T. Hohenester E. (2000). Structure and function of laminin LG modules. Matrix Biol. 19, 309–317. 10.1016/S0945-053X(00)00072-X 10963991

[B70] Tisi D. Talts J. F. Timpl R. Hohenester E. (2000). Structure of the C-terminal laminin G-like domain pair of the laminin α2 chain harbouring binding sites for α-dystroglycan and heparin. EMBO J. 19, 1432–1440. 10.1093/emboj/19.7.1432 10747011 PMC310212

[B71] Utani A. Nomizu M. Matsuura H. Kato K. Kobayashi T. Takeda U. (2001). A unique sequence of the laminin α3 G domain binds to heparin and promotes cell adhesion through syndecan-2 and -4. J. Biol. Chem. 276, 28779–28788. 10.1074/jbc.M101420200 11373281

[B72] Willer T. Inamori K. Venzke D. Harvey C. Morgensen G. Hara Y.. (2014). The glucuronyltransferase B4GAT1 is required for initiation of LARGE-mediated α-dystroglycan functional glycosylation. Elife 3:e03941. 10.7554/eLife.03941 25279699 PMC4227050

[B73] Wizemann H. Garbe J. H. Friedrich M. V. Timpl R. Sasaki T. Hohenester E. (2003). Distinct requirements for heparin and α-dystroglycan binding revealed by structure-based mutagenesis of the laminin α2 LG4-LG5 domain pair. J. Mol. Biol. 332, 635–642. 10.1016/S0022-2836(03)00848-9 12963372

[B74] Wright K. M. Lyon K. A. Leung H. Leahy D. J. Ma L. Ginty D. D. (2012). Dystroglycan organizes axon guidance cue localization and axonal pathfinding. Neuron 76, 931–944. 10.1016/j.neuron.2012.10.009 23217742 PMC3526105

[B75] Xi J. Yan C. Liu W. W. Qiao K. Lin J. Tian X.. (2017). Novel SEA and LG2 Agrin mutations causing congenital Myasthenic syndrome. Orphanet. J. Rare Dis. 12:182. 10.1186/s13023-017-0732-z 29258548 PMC5735900

[B76] Yokoyama F. Suzuki N. Kadoya Y. Utani A. Nakatsuka H. Nishi N. (2005). Bifunctional peptides derived from homologous loop regions in the laminin α chain LG4 modules interact with both α2β1 integrin and syndecan-2. Biochemistry 44, 9581–9589. 10.1021/bi050598t 16008343

[B77] Yoshida-Moriguchi T. Campbell K. P. (2015). Matriglycan: a novel polysaccharide that links dystroglycan to the basement membrane. Glycobiology 25, 702–713. 10.1093/glycob/cwv021 25882296 PMC4453867

[B78] Yu H. Talts J. F. (2003). β1 Integrin and α-dystroglycan binding sites are localized to different laminin-G-domain-like (LG) modules within the laminin α5 chain G domain. Biochem. J. 371, 289–299. 10.1042/bj20021500 12519075 PMC1223287

[B79] Zhang Y. Dai Y. Han J. N. Chen Z. H. Ling L. Pu C. Q.. (2017). A novel AGRN mutation leads to congenital myasthenic syndrome only affecting limb-girdle muscle. Chin. Med. J. 130, 2279–2282. 10.4103/0366-6999.215332 28937031 PMC5634075

[B80] Zhou Y. W. Thomason D. B. Gullberg D. Jarrett H. W. (2006). Binding of laminin α1-chain LG4-5 domain to α-dystroglycan causes tyrosine phosphorylation of syntrophin to initiate Rac1 signaling. Biochemistry 45, 2042–2052. 10.1021/bi0519957 16475793

