# Supplementary material for: Analysis of α-Dystroglycan/LG Domain Binding Modes: Investigating Protein Motifs That Regulate the Affinity of Isolated LG Domains
Source: Front Mol Biosci. 2019 Mar 29;6:18. doi: 10.3389/fmolb.2019.00018 (PMC6450144; doi:10.3389/fmolb.2019.00018)
Supplement: Supplementary file 1 [file Data_Sheet_1.docx]

**Analysis Of α-Dystroglycan/LG Domain Binding Modes: Investigating Protein Motifs That Regulate The Affinity Of Isolated LG Domains**

**Christopher E. Dempsey^1*^, Maria Giulia Bigotti^1^, Josephine C. Adams^1^, Andrea Brancaccio^1,2*^**

^1^School of Biochemistry, University of Bristol, Bristol BS8 1TD, United Kingdom

^2^Istituto di Chimica del Riconoscimento Molecolare - CNR c/o Università Cattolica del Sacro Cuore, I-00168 Roma, Italy

*Authors for Manuscript Correspondence:

Dr. Christopher E. Dempsey

School of Biochemistry,
University of Bristol, Bristol BS8 1TD, United Kingdom

c.dempsey@bristol.ac.uk

Dr. Andrea Brancaccio

Istituto di Chimica del Riconoscimento Molecolare, CNR

Istituto di Biochimica e Biochimica Clinica

Università Cattolica del Sacro Cuore

L.go F. Vito 1 00168-Roma (Italy).
andrea.brancaccio@icrm.cnr.it

**Supplementary material**

**Methods for sequence analysis and homology modelling**

Although the biochemical and structural evidence available has been collected from different tissue sources and species, due to the high degree of inter-species sequence conservation our alignment and modelling is centred on human LG domains

**Protein sequences and analysis**

The LG-domain borders, reported in Table.1, (referring to PS50025 - LAM_G_DOMAIN), have been assessed by feeding all the relevant protein sequences into the InterProScan 5.2 (<https://www.ebi.ac.uk/interpro/>) database for protein sequence analysis and domain classification. All the human protein NCBI accession codes and code names for the corresponding LG domains are reported in Table.1. Fig.S2 provides, in FASTA format, all the primary structures of the human LG domains that were analysed. The LG domain protein sequences were aligned in MUSCLE 3.8 via the resources of EMBL/EBI (<http://www.ebi.ac.uk/Tools/msa>), and are presented in BoxShade 3.21 (<http://www.ch.embnet.org/software/BOX_form.html>). To prepare the phylogenetic diagram LG domain protein sequences were aligned in MAFFT (with re-definition of domain boundaries as necessary for the alignment) at default parameters (<http://mafft.cbrc.jp/alignment/software/>) (Katoh et al., 2005) and the Newick output rendered in iTOL (Letunic and Bork, 2007).

**Homology modelling**

The strong homology within the family of LG domains together with the availability of several high-resolution crystal structures supports the use of homology modelling to identify potential motifs that relate especially to metal ion (Ca^2+^) binding and the structural context of basic residues groupings that may be important for functional interactions. The structures of murine laminin-α2 (LG4-5) (PDB:1QU0), murine laminin-α1 (LG4-5) (PDB:2JD4) and perlecan LG3 (PDB:3SH5) were used since these have bound metal ions (Mg^2+^ in 1QU0 and Ca^2+^ in 2JD4 and 3SH5) that strongly define the coordinating backbone and side chain groups in the Ca^2+^ ion binding pocket. These provided excellent templates for identifying potential Ca^2+^ ion sites in LG domains of unknown structure. In the structural figures, structures deposited in the protein data bank are represented with green backbone ribbons and homology models are represented with blue backbone ribbons.

Homology modelling was performed using Modeller v9.17 (Sali and Blundell, 1993). Modeller was used in two ways as to minimise possible bias in models based on selected input sequence alignments. Sequence alignments were obtained in the first instance from MUSCLE 3.8 as described above and used to construct homology models optimised for these alignments. In addition, we also used the in-built alignment tool in Modeller to create alignments against which homology models were constructed. In most cases small differences in the alignments did not affect the output structures with respect to identification of potential metal ion sites and their coordinating backbone and side chain groups.

To characterize potential metal ion binding sites, homology models were aligned with template crystal structures by RMSD minimization within Pymol and the template metal ion (Mg^2+^ in 1QU0 or Ca^2+^ in 2JD4) “extracted” into the homology model so that coordinating groups in potential metal ion binding sites could be identified. Real or potential Ca^2+^-coordinating backbone and side chain groups are identified for the different structures and models in Table 2 which allows the structural context of potential Ca^2+^ binding to be made with respect to amino acid sequence alignments.

**References to the Methods section**

[**Katoh K, Kuma K, Toh H, Miyata T.** (2005) MAFFT version 5: improvement in accuracy of multiple sequence alignment. *Nucleic Acids Res.* **33**, 511-518.](http://nar.oxfordjournals.org/content/33/2/511.long) doi: 10.1093/nar/gki198

**Letunic I & Bork P**. (2007). Interactive Tree Of Life (iTOL): an online tool for phylogenetic tree display and annotation. *Bioinformatics* **23**, 127-128. doi: 10.1093/bioinformatics/btl529

**Sali, A. & Blundell, T. L** (1993) Comparative modelling by satisfaction of  
spatial restraints. *J. Mol. Biol.* **234**, 779-815. doi: 10.1006/jmbi.1993.1626

**A** **A** **B** **B** **C** **C D**

LNA4_LG2 1 AASYFFDGS-GYAVV**R**DIT**RR**G----**K**FGQVTRFDIEV**R**------TPADNG-----LILL
LNA3_LG2 1 SD**K**NYFEGT-GYARVPTQPHA-----PIPT---FGQTIQ------TTVD**R**G-----LLFF
NRX3A_LG6 1 ATYIFG**K**SG-GLILYTWPAND**R**----PST**R**SDRLAVGFS------TTV**K**DG-----ILVR
NRX3B_LG1 1 ATYIFG**K**SG-GLILYTWPAND**R**----PST**R**SDRLAVGFS------TTV**K**DG-----ILVR
NRX1A_LG6 1 --YIFS**K**GG-GQITY**K**WPPND**R**----PST**R**ADRLAIGFS------TVQ**K**EA-----VLVR
NRX1B_LG1 1 --YIFS**K**GG-GQITY**K**WPPND**R**----PST**R**ADRLAIGFS------TVQ**K**EA-----VLVR
NRX2A_LG6 1 TTYIFG**K**GG-ALITYTWPPND**R**----PST**R**MDRLAVGFS------THQ**R**SA-----VLVR
NRX2B_LG1 1 TTYIFG**K**GG-ALITYTWPPND**R**----PST**R**MDRLAVGFS------THQ**R**SA-----VLVR
LNA4_LG1 1 QVSMMFDGQ-SAVEVHS**R**TSMDDL--**K**AFTSLSLYMKPPV**K**RPELTETADQ-----FILY
LNA3_LG1 1 -PM**R**FNG**K**SGVEVRLPNDLEDL----**K**GYTSLSLFLQRP------NS**R**ENGGTENMFVMY
LNA5_LG1 1 -PM**K**FNG**R**SGVQLRTP**R**DLADL----AAYTALKFYLQGP------EPEPGQGTEDRFVMY
NRX2A_LG4 1 --VLSYDGS-MYMKIMLPNA------MHTEAEDVSLRFM------SQ**R**AYG-----LMMA
NRX1A_LG4 1 --VLSYDGS-MFMKIQLPVV------MHTEAEDVSLRF**R**------SQ**R**AYG-----ILMA
NRX3A_LG4 1 --ILSYDGS-MYMKIIMPMV------MHTEAEDVSFRFM------SQ**R**AYG-----LLVA
LNA4_LG3 1 S**RR**AYFNGQ-SFIASIQ**K**I-------SFFDGFEGGFNF**R**------TLQPNG-----LLFY
LNA3_LG3 1 V**R**SASFS**R**G-GQLSFTDLGL------PPTDHLQASFGFQ------TFQPSG-----ILLD
LNA5_LG3 1 --AMTFHGH-GFLRLALSNVA-----PLTGNVYSGFGFH------SAQDSA-----LLYY
LNA5_LG2 1 TDGSYLDGT-GFARISFDS-------QISTT**K**RFEQEL**R**------LVSYSG-----VLFF
LNA3_LG5 1 -**K**GIYFSEEGGHVVLAHSV-------LLGPEFKLVFSI**R**------P**R**SLTG-----ILIH
LNA5_LG5 1 -AGLFFPGSGGVITLDLPGATL----PDVG---LELEV**R**------PLAVTG-----LIFH
LNA4_LG5 1 -TGTYFSTEGGYVVLDESFNIG----L**K**FE---IAFEV**R**------P**R**SSSG-----TLVH
LNA1_LG5 1 QEGTYFDGS-GYAALV**K**EGY------**K**VQSDVNITLEF**R**------TSSQNG-----VLLG
LNA2_LG5 1 Q**R**GTYFDGT-GFAKAVGGF-------**K**VGLDLLVEFEF**R**------TTTTTG-----VLLG
LNA5_LG4 1 -SYQFGGSLSSHLEFVGILA------**R**H**R**NWPSLSMHVL------P**R**SS**R**G-----LLLF
LNA3_LG4 1 -ALQFGDIPTSHLLF**K**LPQELL**K**---P**R**SQ---FAVDMQ------TTSS**R**G-----LVFH
LNA4_LG4 1 -AYQYGGTANS**R**QEFEHL**K**G------DFGA**K**SQFSIRL**R**------T**R**SSHG-----MIFY
LNA1_LG1 1 **K**VAVSAD**R**D-CIRAYQPQI-------SSTNYNTLTLNV**K**------TQEPDN-----LLFY
LNA2_LG1 1 **K**VSVSSGGD-CIRTY**K**PEI**KK**G----SYNN---IVVNV**K**------TAVADN-----LLFY
LNA1_LG3 1 I**R**SVSFL**K**G-GYIELPP**K**SLS-----PESE---WLVTFA------TTNSSG-----IILA
LNA2_LG3 1 VYTVSFP**K**P-GFVELSPVPID-----VGTE---INLSFS------T**K**NESG-----IILL
NRX2A_LG2 1 --VATF**K**GN-EFFCYDLSHNP-----IQSSTDEITLAF**R**------TLQ**R**NG-----LMLH
NRX1A_LG2 1 --IATF**K**GS-EYFCYDLSQNP-----IQSSSDEITLSF**K**------TLQ**R**NG-----LMLH
NRX3A_LG2 1 --VATF**R**GS-EYLCYDLSQNP-----IQSSSDEITLSF**K**------TWQ**R**NG-----LILH
NRX1A_LG1 1 -GLEFPGAEGQWTRFP**K**WNAC-----CESE---MSFQL**K**------T**R**SA**R**G-----LVLY
NRX2A_LG1 1 -GLEFGGGPGQWARYA**R**WA-------GAASSGELSFSL**R**------TNAT**R**A-----LLLY
NRX3A_LG1 1 -GLEFMGLPNQWARYL**R**WDAS-----T**R**SD---LSFQF**K**------TNVSTG-----LLLY
LNA1_LG2 1 DPSFHFDGS-GYSVVE**K**SLPA-----TVTQ---IIMLFN------TFSPNG-----LLLY
LNA2_LG2 1 --TIQFDGE-GYALVS**R**PI**R**WY----PNIST--VMFKF**R**------TFSSSA-----LLMY
LNA2_LG4 1 -S**K**QFGLS**R**NSHIAIAFDDT------**K**V**K**N**R**LTIELEV**R**------TEAESG-----LLFY
LNA1_LG4 1 -AHQFGLTQNSHFILPFNQS------AV**RKK**LSVELSI**R**------TFASSG-----LIYY
PKC_LG3 1 IEIPQFIG**R**-SYLTYDNPDIL**KR**---VSGS**R**SNVFMRF**K**------TTA**K**DG-----LLLW
**PRL_LG3** 1 QY**GAYF**HDD-G**FLAFPGHV**FSRS---LPEVP**ETIELEVR**------TSTAS**G**-----**LLLW**
SLT3_LG1 1 ITVNFVG**K**D-SYVELASA**K**V**R**-----PQAN---ISLQVA------TD**K**DNG-----ILLY
SLT1_LG1 1 LSVNFVD**R**D-TYLQFTDLQNW-----P**R**AN---ITLQVS------TAEDNG-----ILLY
SLT2_LG1 1 VSVNFIN**K**E-SYLQIPSA**K**V**R**-----PQTN---ITLQIA------TDEDSG-----ILLY
NRX1A_LG5 1 DPVTF**K**T**K**S-SYVALATLQAY-----TSMH---LFFQF**K**------TTSLDG-----LILY
NRX3A_LG5 1 DPVTF**K**T**K**S-SYLSLATLQAY-----TSMH---LFFQF**K**------TTSPDG-----FILF
NRX2A_LG5 1 DPVTF**K**S**R**S-SYLALATLQAY-----ASMH---LFFQF**K**------TTAPDG-----LLLF
PKC_LG1 1 IQYPQFFGH-SYVTFEPL**K**-------NSYQAFQITLEF**R**------AEAEDG-----LLLY
PKC_LG2 1 -IPQF**R**ESL**R**SYAATPWPLEPQ----HYLSFMEFEITF**R**------PDSGDG-----VLLY
NRX2A_LG3 1 DPVTFESPE-AFVALP**R**WSA**K**-----**R**TGS---ISLDF**R**------TTEPNG-----LLLF
NRX1A_LG3 1 DPITFETPE-SFISLP**K**WNA**K**-----**K**TGS---ISFDF**R**------TTEPNG-----LILF
NRX3A_LG3 1 DPINFETPE-AYISLP**K**WNT**K**-----**R**MGS---ISFDF**R**------TTEPNG-----LILF
AGR_LG3 1 NAVTESELA-NEIPVPETLDSGALHE**K**ALQSNHFELSL**R**------TEATQG-----LVLW
PRL_LG1 1 --VPYFTQT-PYSFLPLPTI**K**-----DAY**RK**FEIKITF**R**------PDSADG-----MLLY
PRL_LG2 1 -TTPSLSGAGSYLALPALT-------NTHHEL**R**LDVEF**K**------PLAPDG-----VLLF
AGR_LG2 1 PFLADFNGF-SHLEL**R**GLHTFA**R**---DLGE**K**MALEVVFL------A**R**GPS**G**-----LLLY
AGR_LG1 1 APVPAFEG**R**-SFLAFPTL**R**AY-----HTL**R**---LALEF**R**------ALEPQG-----LLLY
 **AAAA BBBBhhhh** **CCCCCCCC D** **DDDD** **D E E F F G G**

LNA4_LG2 45 M--------------VNGSMFFRLEM-RNGYLHVFYDFGFSG---------GPVHLEDTL
LNA3_LG2 41 A--------------ENGD**R**FISLNI-EDG**K**LMVRY**K**LN--S---------EL---PKE**R**
NRX3A_LG6 45 I-----------DSAPGLGDFLQLHI-EQG**K**IGVVFNIG--T---------VD---ISI**K**
NRX3B_LG1 45 I-----------DSAPGLGDFLQLHI-EQG**K**IGVVFNIG--T---------VD---ISI**K**
NRX1A_LG6 43 V-----------DSSSGLGDYLELHI-HQG**K**IGVKFNVG--T---------DD---IAIE
NRX1B_LG1 43 V-----------DSSSGLGDYLELHI-HQG**K**IGVKFNVG--T---------DD---IAIE
NRX2A_LG6 45 V-----------DSASGLGDYLQLHI-DQGTVGVIFNVG--T---------DD---ITID
NRX2B_LG1 45 V-----------DSASGLGDYLQLHI-DQGTVGVIFNVG--T---------DD---ITID
LNA4_LG1 53 L-----------GS**K**NA**KK**EYMGLAI-**K**NDNLVYVYNLG--T---------KD---VEIP
LNA3_LG1 50 L-----------GN**K**DAS**R**DYIGMAV-VDGQLTCVYNLGD**R**E---------AELQVDQIL
LNA5_LG1 50 M-----------GS**R**QATGDYMGVSL-**R**DK**K**VHWVYQLGE-A---------GP---AVLS
NRX2A_LG4 41 T------------TS**R**ESADTLRLEL-DGGQMKLTVNLD--CL**R**VGCAPS**K**GP---ETLF
NRX1A_LG4 41 T------------TS**R**DSADTLRLEL-DAG**R**VKLTVNLD--CI**R**INCNSS**K**GP---ETLF
NRX3A_LG4 41 T------------TS**R**DSADTLRLEL-DGG**R**VKLMVNLD--CI**R**INCNSS**K**GP---ETLY
LNA4_LG3 42 Y--------------ASGSDVFSISL-DNGTVIMDV**K**--------------GI---**K**VQS
LNA3_LG3 43 H--------------QTWT**R**NLQVTL-EDGYIELSTSDS--G---------GP----IF**K**
LNA5_LG3 42 **R**--------------ASPDGLCQVSL-QQG**R**VSLQL---------------L**R**---TEV**K**
LNA5_LG2 42 L--------------**K**QQSQFLCLAV-QEGSLVLLYDFG--A---------GL**KK**AVPLQ
LNA3_LG5 42 I-------------GSQPG**K**HLCVYL-EAG**K**VTASMDSG--A---------GG---TSTS
LNA5_LG5 42 L------------GQA**R**TPPYLQLQV-TEKQVLLRADDG--A---------GE---FSTS
LNA4_LG5 42 G-------------HSVNGEYLNVHM-**K**NGQVIVKVNNG--I---------**R**D---FSTS
LNA1_LG5 43 I-------------STA**K**VDAIGLEL-VDG**K**VLFHVNNG--A---------G**R**---ITAA
LNA2_LG5 42 I-------------SSQ**K**MDGMGIEM-IDE**K**LMFHVDNG--A---------G**R**---FTAV
LNA5_LG4 43 T-----------ARL**R**PGSPSLALFL-SNGHFVAQMEGL--G---------T**R**---LRAQ
LNA3_LG4 43 T--------------GT**K**NSFMALYL-S**K**G**R**LVFALGTD--G---------**KK**---LRI**K**
LNA4_LG4 43 V------------SDQEENDFMTLFL-AHG**R**LVYMFNVG--H---------**KK**---LKI**R**
LNA1_LG1 42 L------------GSSTASDFLAVEM-**RR**G**R**VAFLWDLG--S---------GS---TRLE
LNA2_LG1 42 L------------GSA**K**FIDFLAIEM-**RK**G**K**VSFLWDVG--S---------GV---GRVE
LNA1_LG3 41 A--LGGDVE**KR**GD**R**EEAHVPFFSVML-IGGNIEVHVNPG--D---------GTGL**RK**ALL
LNA2_LG3 41 G-SGGTPAPP**RRKRR**QTGQAYYAILL-N**R**G**R**LEVHLSTG--A---------RTM**RK**IVIR
NRX2A_LG2 42 T--------------G**K**SADYVNLSL-**K**SGAVWLVINLG--S---------GA--FEALV
NRX1A_LG2 42 T--------------G**K**SADYVNLAL-**K**NGAVSLVINLG--S---------GA--FEALV
NRX3A_LG2 42 T--------------G**K**SADYVNLAL-**K**DGAVSLVINLG--S---------GA--FEAIV
NRX1A_LG1 41 F------------DDEGFCDFLELILT**R**GG**R**LQLSFSIF--C---------AE---PATL
NRX2A_LG1 42 L------------DDGGDCDFLELLL-VDG**R**LRLRFTLS--CA--------EP---ATLQ
NRX3A_LG1 41 L------------DDGGVCDFLCLSL-VDG**R**VQLRFSMD--C---------AE---TAVL
LNA1_LG2 41 L------------GSYGT**K**DFLSIEL-F**R**G**R**VKVMTDLG--S---------GP---ITLL
LNA2_LG2 41 L------------AT**R**DL**R**DFMSVEL-TDGHIKVSYDLG--S---------GM---ASVV
LNA2_LG4 43 M------------A**R**INHADFATVQL-**R**NGLPYFSYDLG--S---------GD---THTM
LNA1_LG4 43 M------------AHQNQADYAVLQL-HGG**R**LHFMFDLG--**K**---------G**R**---TKVS
PKC_LG3 46 **R**---------GDSPM**R**PNSDFISLGL-**R**DGALVFSYNLG--S---------GV---ASIM
**PRL_LG3** 46 **Q**-------GVEVGEAGQGK**DFISLGL**-QDG**HLVFRYQL**G--S---------GE---**ARLV**
SLT3_LG1 41 **K**---------------GDNDPLALEL-YQGHVRLVYDSL--S---------SP---PTTV
SLT1_LG1 41 N---------------GDNDHIAVEL-YQGHVRVSYDPG--S---------YP---SSAI
SLT2_LG1 41 **K**---------------GD**K**DHIAVEL-Y**R**G**R**VRASYDTG--S---------HP---ASAI
NRX1A_LG5 41 N-------------SGDGNDFIVVEL-V**K**GYLHYVFDLG--N---------GA---NLI**K**
NRX3A_LG5 41 N-------------SGDGNDFIAVEL-V**K**GYIHYVFDLG--N---------GP---NVI**K**
NRX2A_LG5 41 N-------------SGNGNDFIVIEL-V**K**GYIHYVFDLG--N---------GP---SLM**K**
PKC_LG1 42 C----------GENEHG**R**GDFMSLAI-I**R**RSLQFRFNCG--T---------GV---AIIV
PKC_LG2 45 S------------YDTGS**K**DFLSINL-AGGHVEFRFDCG--S---------GT---GVL**R**
NRX2A_LG3 41 SQG**RR**AGGGAGSHSSAQ**R**ADYFAMEL-LDGHLYLLLDMG--S---------GG---IKL**R**
NRX1A_LG3 41 SHG**K**P**R**HQ**K**DA**K**HPQMI**K**VDFFAIEM-LDGHLYLLLDMG--S---------GT---IKI**K**
NRX3A_LG3 41 THG**K**PQE**RK**DA**R**SQ**K**NT**K**VDFFAVEL-LDGNLYLLLDMG--S---------GT---IKV**K**
AGR_LG3 49 S-----------G**K**ATERADYVALAI-VDGHLQLSYNLG--S---------QP---VVL**R**
PRL_LG1 42 N-GQ**KR**VPGSPTNLAN**R**QPDFISFGL-VGG**R**PEFRFDAG--S---------GM---ATI**R**
PRL_LG2 42 S----------GG**K**SGPVEDFVSLAM-VGGHLEFRYELG--S---------GL---AVL**R**
AGR_LG2 46 N----------GQ**K**TDG**K**GDFVSLAL-**R**D**RR**LEFRYDLG--**K**---------**G**A---AVI**R**
AGR_LG1 41 N------------GNA**R**G**K**DFLALAL-LDG**R**VQLRFDTG--S---------GP---AVLT
 **D *EEEEEE**  **FFFFFFF* GGGG** **H H I I**

LNA4_LG2 81 **KK**AQ----INDA**K**YHEISIIY-HND**K**K---------------MILVV------D-----**R**
LNA3_LG2 72 GVGD-A--INNG**R**DHSIQIKIGKLQ**K**R---------------MWINV------DVQ---N
NRX3A_LG6 79 EE**R**T-P--VNDG**K**YHVVRFT--RNGGN---------------ATLQV------DN----W
NRX3B_LG1 79 EE**R**T-P--VNDG**K**YHVVRFT--RNGGN---------------ATLQV------DN----W
NRX1A_LG6 77 ESNA-I--INDG**K**YHVVRFT--RSGGN---------------ATLQV------DS----W
NRX1B_LG1 77 ESNA-I--INDG**K**YHVVRFT--RSGGN---------------ATLQV------DS----W
NRX2A_LG6 79 EPNA-I--VSDG**K**YHVVRFT--RSGGN---------------ATLQV------DS----W
NRX2B_LG1 79 EPNA-I--VSDG**K**YHVVRFT--RSGGN---------------ATLQV------DS----W
LNA4_LG1 87 LDS**K**-PVSSWPAYFSIVKIE--RVG**K**H---------------GKVFLTVPSLSSTAEE**K**F
LNA3_LG1 89 TKSE----TKEAVMDRVKFQ--RIYQF---------------ARLNY-----T**K**GATSS**K**
LNA5_LG1 85 IDED----IGEQ-FAAVSLD--RTLQF---------------GHMSVTVE**R**QMIQE---T
NRX2A_LG4 83 AGH**K**----LNDNEWHTVRVV--R**R**G**K**S---------------LQLSV------DN----V
NRX1A_LG4 83 AGYN----LNDNEWHTVRVV--R**R**G**K**S---------------LKLTV------DDQ---Q
NRX3A_LG4 83 AGQ**K**----LNDNEWHTVRVV--R**R**G**K**S---------------LKLTV------DD----D
LNA4_LG3 70 VD**K**Q----YNDGLSHFVISS--VSPT**R**---------------YELIV------D**K**S---**R**
LNA3_LG3 73 SPQT----YMDGLLHYVSVI--SDNSG---------------LRLLI------DDQ----
LNA5_LG3 69 TQAG----FADGAPHYVAFY--SNATG---------------VWLYV------DDQ---L
LNA5_LG2 76 PPPPLT--SAS**K**AIQVFLLG--GS**RK**R---------------VLVRV------------E
LNA3_LG5 74 VTP**K**QS--LCDGQWHSVAVT--I**K**QHI---------------LHLEL------DTD---S
LNA5_LG5 75 VT**R**PSV--LCDGQWHRLAVM--KSGNV---------------LRLEV------DAQ---S
LNA4_LG5 74 VTP**K**QS--LCDG**R**WHRITVI--RDSNV---------------VQLDV------DS----E
LNA1_LG5 75 YEP**K**TATVLCDG**K**WHTLQAN--KS**K**H**R**---------------ITLIV------DGN---A
LNA2_LG5 74 YDAGVPGHLCDGQWHKVTAN--KI**K**H**R**---------------IELTV------DGN---Q
LNA5_LG4 77 S**R**Q**R**----S**R**PG**R**WHKVSVR--WE**K**N**R**---------------ILLVT------DGA---**R**
LNA3_LG4 74 S**K**E**K**----CNDG**K**WHTVVFG--HDGE**K**---------------GRLVV------DGL---**R**
LNA4_LG4 76 SQE**K**----YNDGLWHDVIFI--RE**R**SS---------------GRLVI------DGL---**R**
LNA1_LG1 75 FPDF-P--IDDN**R**WHSIHVA--RFGNI---------------GSLSV**K**EMSSNQ**K**S---P
LNA2_LG1 75 YPDL-T--IDDSYWYRIVAS--RTG**R**N---------------GTISV**R**ALDGP**K**ASIVPS
LNA1_LG3 87 HAPTGT--CSDGQAHSISLV--RN**RR**I---------------ITVQL------DEN---N
LNA2_LG3 88 PEPN-L--FHDG**R**EHSVHVE--RT**R**GI---------------FTVQV------DE----N
NRX2A_LG2 74 EPVNG**K**--FNDNAWHDVRVT--RNL**R**QHAGIGHAMVNKLHYLVTISV------DGI---L
NRX1A_LG2 74 EPVNG**K**--FNDNAWHDVKVT--RNL**R**Q---------------VTISV------DGI---L
NRX3A_LG2 74 EPVNG**K**--FNDNAWHDVKVT--RNL**R**Q---------------VTISV------DGI---L
NRX1A_LG1 75 LADT-P--VNDGAWHSVRIR--RQF**R**N---------------TTLFI------DQV---E
NRX2A_LG1 76 LDTP----VADD**R**WHMVLLT--RDA**R**R---------------TALAV------DG----E
NRX3A_LG1 74 SN**K**Q----VNDSSWHFLMVS--RD**R**LR---------------TVLML------DG----E
LNA1_LG2 74 TD**RR**----YNNGTWYKIAFQ--RN**RK**Q---------------GVLAVIDAYNTSN**K**---E
LNA2_LG2 74 SNQN----HNDG**K**W**K**SFTLS--RIQ**K**Q---------------ANISIVDI-DTNQE---E
LNA2_LG4 76 IPT**K**----INDGQWHKIKIM--RS**K**QE---------------GILYV------DGA---S
LNA1_LG4 76 HPAL----LSDG**K**WHTVKTD--YV**KRK**---------------GFITV------DG**R**---E
PKC_LG3 82 VNGS----FNDG**R**WHRVKAV--RDGQS---------------GKITV------DDY---G
**PRL_LG3** 84 SEDP----INDGEW**HRVTAL**--**RE**G**RR**---------------**GSIQV**------DGE---E
SLT3_LG1 71 YSVE-T--VNDGQFHSVELV--TLNQT---------------LNLVV------D**K**G---T
SLT1_LG1 71 YSAE-T--INDGQFHTVELV--AFDQM---------------VNLSI------DGG---S
SLT2_LG1 71 YSVE-T--INDGNFHIVELL--ALDQS---------------LSLSV------DGG---N
NRX1A_LG5 73 GSSN**K**P--LNDNQWHNVMIS--RDTSN--------------LHTVKI------D-----T
NRX3A_LG5 73 GNSD**R**P--LNDNQWHNVVIT--RDNSN----------------THSL------**K**VD---T
NRX2A_LG5 73 GNSD**K**P--VNDNQWHNVVVS--RDPGN--------------VHTLKI------DS----**R**
PKC_LG1 77 SET**K**----IKLGGWHTVMLY--RDGLN---------------GLLQL------NNG---T
PKC_LG2 78 SEDP----LTLGNWHELRVS--RTA**K**N---------------GILQV------D**K**Q---**K**
NRX2A_LG3 86 ASS**R**-**K**--VNDGEWCHVDFQ--RDG**RK**---------------GSISV------NS**R**---S
NRX1A_LG3 86 ALL**K**-**K**--VNDGEWYHVDFQ--RDG**R**S---------------GTISV------NTL---**R**
NRX3A_LG3 86 ATQ**K**-**K**--ANDGEWYHVDIQ--RDG**R**S---------------GTISV------NS**R**---**R**
AGR_LG3 83 STVP----VNTNRWLRVVAH--REQ**R**E---------------GSLQV------GNE---A
PRL_LG1 86 HPTP----LALGHFHTVTLL--RSLTQ---------------GSLIV------GDL---A
PRL_LG2 77 SAEP----LALGRWHRVSAE--RLN**K**D---------------GSLRV------NGG---**R**
AGR_LG2 81 SREP----VTLGAWTR**V**SLE--RNG**RK**---------------GALRV------GDG---P
AGR_LG1 74 SAVP----VEPGQWHRLELS--RHW**RR**---------------GTLSV------DGE---T
 **HHHHHH HH I IIIII** **J J K** **K**

LNA4_LG2 110 **R**HV--KSMDN------------------------------E**K**M**K**IPF--TD-----IYIG
LNA3_LG2 105 TII-------------------------------------DGEVFDF--ST-----YYLG
NRX3A_LG6 109 PVN--EHYPT------------------------------GRQLTIFNTQAQ----IAIG
NRX3B_LG1 109 PVN--EHYPT------------------------------GRQLTIFNTQAQ----IAIG
NRX1A_LG6 107 PVI--E**R**YPA------------------------------GRQLTIFNSQAT----IIIG
NRX1B_LG1 107 PVI--E**R**YPA------------------------------GRQLTIFNSQAT----IIIG
NRX2A_LG6 109 PVN--E**R**YPAGNFDNE**R**LAIA**R**Q**R**IPY**R**LG**R**VVDEWLLD**K**G**R**QLTIFNSQAA----I**K**IG
NRX2B_LG1 109 PVN--E**R**YPAGNFDNE**R**LAIA**R**Q**R**IPY**R**LG**R**VVDEWLLD**K**G**R**QLTIFNSQAA----I**K**IG
LNA4_LG1 129 I**K**K--GEFSG------------------------------DDSLLDLDPEDTV---FYVG
LNA3_LG1 123 PETPGVYDMD------------------------------GRNSNTLLNLDPENVVFYVG
LNA5_LG1 120 **K**GD--TVAPG------------------------------AEGLLNL**R**PDDFV---FYVG
NRX2A_LG4 112 TVE--GQMAG------------------------------AHM**R**LEF--HN-----IETG
NRX1A_LG4 113 AMT--GQMAG------------------------------DHT**R**LEF--HN-----IETG
NRX3A_LG4 112 VAE--GTMVG------------------------------DHT**R**LEF--HN-----IETG
LNA4_LG3 100 VGS--KNPT**K**------------------------------G**K**IEQTQASE**KK**----FYFG
LNA3_LG3 102 -----LL**R**NS------------------------------**KR**L**K**HISSSRQS----L**R**LG
LNA5_LG3 99 QQM--KPH**R**G------------------------------PPPELQPQPEGPP**R**--LLLG
LNA5_LG2 105 **R**AT--VYSVE------------------------------QDNDLEL--ADA----YYLG
LNA3_LG5 106 SYT---AGQI------------------------------PFPPAST--QEP----LHLG
LNA5_LG5 107 NHT---VGPL------------------------------LAAAAGA--PAP----LYLG
LNA4_LG5 105 VNH--VVGPL------------------------------NP**K**PIDH--**R**EP----VFVG
LNA1_LG5 109 VGA--ESPHT------------------------------QSTSVDT--NNP----IYVG
LNA2_LG5 108 VEA--QSPNP------------------------------ASTSADT--NDP----VFVG
LNA5_LG4 107 AWS--QEGPH-----------------------------**R**QHQGAEHPQPHT----LFVG
LNA3_LG4 104 A**R**E--GSLPG-------------------------------NSTISI--**R**AP----VYLG
LNA4_LG4 106 VLE--ESLPP------------------------------TEATW**K**I--**K**GP----IYLG
LNA1_LG1 112 T**K**T--SKSPG------------------------------TANVLDVNNSTL----MFVG
LNA2_LG1 115 THH--STSPP------------------------------GYTILDVDANAM----LFVG
LNA1_LG3 119 PVEMKLGTLV------------------------------ES**R**TINV--SN-----LYVG
LNA2_LG3 118 R**R**Y--MQNLT------------------------------VEQPIEV--**KK**-----LFVG
NRX2A_LG2 121 TTT--GYTQE------------------------------DYTMLGS--DDF----FYIG
NRX1A_LG2 106 TTT--GYTQE------------------------------DYTMLGS--DDF----FYVG
NRX3A_LG2 106 TTT--GYTQE------------------------------DYTMLGS--DDF----FYVG
NRX1A_LG1 106 A**K**W--VEV**K**S------------------------------**KRR**DMTV--FSG----LFVG
NRX2A_LG1 105 A**R**A--AEV**R**S------------------------------**KRR**EMQV--ASD----LFVG
NRX3A_LG1 103 GQS--GELQP------------------------------Q**R**PYMDV--VSD----LFLG
LNA1_LG2 110 T**K**Q--GETPG------------------------------ASSDLN**R**LD**K**DP----IYVG
LNA2_LG2 109 NIA--TSSSG------------------------------NNFGLDL**K**ADD**K**----IYFG
LNA2_LG4 106 N**R**T---ISP**K**------------------------------**K**ADILDV--VGM----LYVG
LNA1_LG4 106 SPM--VTVVG------------------------------DGTMLDV--EGL----FYLG
PKC_LG3 112 A**R**T--GKSPG------------------------------MM**R**QLNI--NGA----LYVG
**PRL_LG3** 114 L**VS--GR**SPG------------------------------PNV**A**V**N**A--KGS----**VYI**G
SLT3_LG1 102 P**K**S--LG**K**LQ------------------------------**K**QPAVGI--NSP----LYLG
SLT1_LG1 102 PMT--MDNFG------------------------------**K**HYTLNS--EAP----LYVG
SLT2_LG1 102 P**K**I--ITNLS------------------------------**K**QSTLNF--DSP----LYVG
NRX1A_LG5 104 **K**IT--TQITA------------------------------GA**R**NLDL--**K**SD----LYIG
NRX3A_LG5 104 **K**VV--TQVIN------------------------------GA**K**NLDL--**K**GD----LYMA
NRX2A_LG5 105 TVT---QHSN------------------------------GA**R**NLDL--**K**GE----LYIG
PKC_LG1 107 PVT--GQSQG------------------------------QYS**K**ITF--**R**TP----LYLG
PKC_LG2 108 IVE--GMAEG------------------------------GFTQIKC--NTD----IFIG
NRX2A_LG3 117 TPF---LATG------------------------------DSEILDL--ESE----LYLG
NRX1A_LG3 117 TPY---TAPG------------------------------ESEILDL--DDE----LYLG
NRX3A_LG3 117 TPF---TASG------------------------------ESEILDL--EGD----MYLG
AGR_LG3 113 PVT--GSSPL------------------------------GATQLDT--DGA----LWLG
PRL_LG1 116 PVN--GTSQG------------------------------**K**FQGLDL--NEE----LYLG
PRL_LG2 107 PVL--RSSPG------------------------------**K**SQGLNL--HTL----LYLG
AGR_LG2 111 **R**VL--GESPV------------------------------PHTVLNL--**K**EP----LYVG
AGR_LG1 104 PVL--GESPS------------------------------GTDGLNL--DTD----LFVG
 **JJ** **JJ** ***** *** KKK** **L L M M**

LNA4_LG2 131 GAPPEILQS**R**AL**R**A---HLPLDINF**R**GCMKGFQFQ**KK**---DFNL-----LEQTETLGVGY
LNA3_LG2 121 GIPI-AI**R**E**R**-------FNISTPAF**R**GCMKNLKKTSG---VV**R**LN--------DTVGVT**K**
NRX3A_LG6 133 G**K**D**K**-----------------G**R**LFQGQLSGLYYDGL---**K**VLNMAAENNPNI**K**INGSV-
NRX3B_LG1 133 G**K**D**K**-----------------G**R**LFQGQLSGLYYDGL---**K**VLNMAAENNPNI**K**INGSV-
NRX1A_LG6 131 G**K**EQ-----------------GQPFQGQLSGLYYNGL---**K**VLNMAAENDANIAIVGNV-
NRX1B_LG1 131 G**K**EQ-----------------GQPFQGQLSGLYYNGL---**K**VLNMAAENDANIAIVGNV-
NRX2A_LG6 163 G**R**DQ-----------------G**R**PFQGQVSGLYYNGL---**K**VLALAAESDPNV**R**TEGHL**R**
NRX2B_LG1 163 G**R**DQ-----------------G**R**PFQGQVSGLYYNGL---**K**VLALAAESDPNV**R**TEGHL**R**
LNA4_LG1 154 GVPS-NF**K**LP-------TSLNLPGFVGCLELATLNND---VISLYNF**K**HIYNMDPSTSVP
LNA3_LG1 153 GYPP-DF**K**LP-------SRLSFPPY**K**GCIELDDLNEN---VLSLYNF**KK**TFNLNTTEVEP
LNA5_LG1 145 GYPS-TFTPP-------PLL**R**FPGY**R**GCIEMDTLNEE---VVSLYNFE**R**TFQLDTAVDRP
NRX2A_LG4 133 IMTE-**RR**F---------ISVVPSNFIGHLSGLVFNGQ---PY-------MDQC**K**DGDITY
NRX1A_LG4 134 IITE-**RR**Y---------LSSVPSNFIGHLQSLTFNGM---AY-------IDLC**K**NGDIDY
NRX3A_LG4 133 IMTE-**KR**Y---------ISVVPSSFIGHLQSLMFNGL---LY-------IDLC**K**NGDIDY
LNA4_LG3 124 GSP--------------ISAQYANFTGCISNAYFT**R**V---D**R**DVEVEDFQ**R**YTE**K**VHTSL
LNA3_LG3 123 G---------------------SNFEGCISNVFVQ**R**L---SLSPEVLDLTSNSL**KR**DVSL
LNA5_LG3 125 GLPE--------------SGTIYNFSGCISNVFVQ**R**L---LGPQ**R**VFDLQQNLGSVNVST
LNA5_LG2 127 GVPP-DQLPPSL**RR**---LFPTGGSV**R**GCVKGIKALG**K**---YVDL------**KR**LNTTGVSA
LNA3_LG5 127 GAPA-NLTTL-------RIPVW**K**SFFGCLRNIHVNHI---PVPV-----TEALEVQGPVS
LNA5_LG5 128 GLPE-PMA---------VQPWPPAYCGCMRRLAVN**R**S---PVAM-----T**R**SVEVHGAVG
LNA4_LG5 127 GVPE-SLLTP-------RLAPS**K**PFTGCIRHFVIDGH---PVSF-----S**K**AALVSGAVS
LNA1_LG5 131 GYPA-GV**K**Q**K**-------CL**R**SQTSF**R**GCLRKLALI**K**S---PQ-VQSFDFS**R**AFELHGVFL
LNA2_LG5 130 GFPD-DL**K**QF-------GLTTSIPF**R**GCIRSLKLT**K**G---TG**K**PLEVNFA**K**ALEL**R**GVQP
LNA5_LG4 132 GLPA-SSHSS-------**K**LPVTVGFSGCVKRLRLHG**R**---PL-------GAPT**R**MAGVTP
LNA3_LG4 125 SPPS-G**K**P---------**K**SLPTNSFVGCLKNFQLDS**K**---PL-------YTPSSSFGVSS
LNA4_LG4 128 GVAP-G**K**AV**K**NV-----QINSIYSFSGCLSNLQLNGA---SI-------TSASQTFSVTP
LNA1_LG1 136 GLGG-QI**KK**S-------PAV**K**VTHF**K**GCLGEAFLNG**K**---SIGL-----WNYIE**R**EGKC**R**
LNA2_LG1 139 GLTG-**K**L**KK**A-------DAV**R**VITFTGCMGETYFDN**K**---PIGL-----WNF**R**E**K**EGDC**K**
LNA1_LG3 142 GIPE-GEGTS-------LLTM**RR**SFHGCIKNLIFNLE---LLDF-----NSAVGHEQVDL
LNA2_LG3 139 GAPP-EFQPS-------PL**R**NIPPFEGCIWNLVINSV---PMDF-----A**R**PVSF**K**NADI
NRX2A_LG2 143 GSPN-TADLP-------GSPVSNNFMGCLKDVVYKNN---DF**K**LELS**R**LA**K**EGDP**K**MKLQ
NRX1A_LG2 128 GSPS-TADLP-------GSPVSNNFMGCLKEVVYKNN---DV**R**LELS**R**LA**K**QGDP**K**MKIH
NRX3A_LG2 128 GSPS-TADLP-------GSPVSNNFMGCLKEVVYKNN---DI**R**LELS**R**LA**R**IADT**K**MKIY
NRX1A_LG1 128 GLPP-EL**R**AAAL**K**LTLASV**R**E**R**EPF**K**GWIRDVRVNSSQVLPVDSGEV**K**LDDEPPNSGGGS
NRX2A_LG1 127 GIPP-DV**R**LSALTLS--TV**K**YEPPF**R**GLLANLKLGER---PPALLG---SQGL**R**GATADP
NRX3A_LG1 125 GVPT-DI**R**PSALTLD--GVQAMPGF**K**GLILDLKYGNS---EP**R**LLG---S**R**GVQMDAEGP
LNA1_LG2 134 GLP**R**-S**R**VV**R**-------**R**GVTT**K**SFVGCIKNLEIS**R**S---TFDL-------L**R**NSYGVR**K**
LNA2_LG2 133 GLPT-L**R**NLSM**K**A**R**---PEVNL**KK**YSGCLKDIEIS**R**T---PYNI-----LSSPDYVGVT**K**
LNA2_LG4 127 GLPI-NYTT**RR**------IGPVTYSIDGCVRNLHMAEA---PADL-----EQPTSSFHVGT
LNA1_LG4 128 GLPS-QYQA**RK**------IGNITHSIPACIGDVTVNS**K**---QLD**K**-----DSPVSAFTVN**R**
PKC_LG3 134 GM**K**EIALHT--------N**R**QYM**R**GLVGCISHFTL------STDYHISLVEDAVDG**K**NINT
**PRL_LG3** 136 GAP**D**-**VATLT**-------GG**R**FSSGIT**GCVK**NL**VLHS**A---**R**PGAPPPQPLDLQHRA**QAGA**
SLT3_LG1 124 GIPT-STGLSAL**R**QG--TD**R**PLGGFHGCIHEVRINNE---LQDF**K**----ALPPQSLGVSP
SLT1_LG1 124 GMPV-DVNSAAF**R**LW--QILNGTGFHGCIRNLYINNE---LQDF-----T**K**TQM**K**PGVVP
SLT2_LG1 124 GMPG-**K**SNVASL**R**QA--PGQNGTSFHGCIRNLYINSE---LQDF-----Q**K**VPMQTGILP
NRX1A_LG5 126 GVA**K**-ETY**K**SLP**K**----LVHA**K**EGFQGCLASVDLNG**R**---LPDLI----SDALFCNGQIE
NRX3A_LG5 126 GLAQ-GMYSNLP**K**----LVAS**R**DGFQGCLASVDLNG**R**---LPDLI----NDALH**R**SGQIE
NRX2A_LG5 126 GLS**K**-NMFSNLP**K**----LVAS**R**DGFQGCLASVDLNG**R**---LPDLI----ADALH**R**IGQVE
PKC_LG1 129 GAPSAYWLV**R**-------ATGTN**R**GFQGCVQSLAVNG**R**---**R**IDM**R**PWPLG**K**ALSGADVGE
PKC_LG2 130 GVPNYDDV**KK**-------NSGVL**K**PFSGSIQKIILND**R**---TIHV**K**-HDFTSGVNVENAAH
NRX2A_LG3 138 GLPEGG**R**VDLPLPPEVWTAAL**R**AGYVGCVRDLFIDG**R**---S**R**DL**R**GL--AEAQGAVGVAP
NRX1A_LG3 138 GLPE-N**K**AGLVFPTEVWTALLNYGYVGCIRDLFIDGQ---S**K**DI**R**QM--AEVQSTAGVKP
NRX3A_LG3 138 GLPE-N**R**AGLILPTELWTAMLNYGYVGCIRDLFIDG**R**---S**K**NI**R**QL--AEMQNAAGVKS
AGR_LG3 135 GLPE-LPVGPAL-----P**K**AYGTGFVGCLRDVVVG**R**H---PLHLL----EDAVT**K**PELRP
PRL_LG1 138 GYPD-YGAIP-------**K**AGLSSGFIGCVRELRIQGE---EIVFH----DLNLTAHGISH
PRL_LG2 129 GVEP-SVPLSP------ATNMSAHF**R**GCVGEVSVNG**K**---**R**LDLT----YSFLGSQGIGQ
AGR_LG2 133 G**A**PD-FS**K**LA**R**------AAAVSSGFDGAIQLVSLGG**R**----QLLTPEHVL**R**QVDVTSFAG
AGR_LG1 126 GVPE-DQAAVALE----**R**TFVGAGL**R**GCIRLLDVNNQ---**R**LELGIGP-GAATRGSGVGE
 **h** **hhhhh** **LLLL LLLL MMMM** LNA4_LG2 180 -----GC
LNA3_LG2 162 -----**K**C
NRX3A_LG6 -------
NRX3B_LG1 -------
NRX1A_LG6 -------
NRX1B_LG1 -------
NRX2A_LG6 203 LVGEGPS
NRX2B_LG1 203 LVGEGPS
LNA4_LG1 203 ------C
LNA3_LG1 202 ------C
LNA5_LG1 194 ------C
NRX2A_LG4 173 ------C
NRX1A_LG4 174 ------C
NRX3A_LG4 173 ------C
LNA4_LG3 167 ----YEC
LNA3_LG3 159 ----GGC
LNA5_LG3 168 -----GC
LNA5_LG2 174 -----GC
LNA3_LG5 171 ---LNGC
LNA5_LG5 170 ---ASGC
LNA4_LG5 171 ---INSC
LNA1_LG5 179 ----HSC
LNA2_LG5 179 ----VSC
LNA5_LG4 174 ------C
LNA3_LG4 165 ------C
LNA4_LG4 172 ------C
LNA1_LG1 180 -----GC
LNA2_LG1 183 -----GC
LNA1_LG3 186 ----DTC
LNA2_LG3 183 ----G**R**C
NRX2A_LG2 192 GDLSF**R**C
NRX1A_LG2 177 GVVAF**K**C
NRX3A_LG2 177 GEVVF**K**C
NRX1A_LG1 187 -----PC
NRX2A_LG1 178 -----LC
NRX3A_LG1 176 ------C
LNA1_LG2 176 -----GC
LNA2_LG2 181 -----GC
LNA2_LG4 172 ------C
LNA1_LG4 173 ------C
PKC_LG3 180 ------C
**PRL_LG3** 185 --N**TRP**C
SLT3_LG1 174 -----GC
SLT1_LG1 173 -----GC
SLT2_LG1 173 -----GC
NRX1A_LG5 174 ----RGC
NRX3A_LG5 174 ----RGC
NRX2A_LG5 174 ----RGC
PKC_LG1 179 ------C
PKC_LG2 179 -----PC
NRX2A_LG3 193 -----FC
NRX1A_LG3 192 -----SC
NRX3A_LG3 192 -----SC
AGR_LG3 182 ------C
PRL_LG1 183 ---CPTC
PRL_LG2 175 CYDSSPC
AGR_LG2 182 ----HPC
AGR_LG1 177 ------C
 **NNN**

**Figure S1: Multiple alignment of all the human LG domains under analysis.** A multiple sequence alignment of all the human LG domains considered was prepared in MUSCLE 3.8. LG3 of perlecan (3SH4/3SH5) was used as a register for relevant secondary structure elements (the 14 β-strands, from A to N, and the 2 helices): β-strands, from A to N (red), α-helices (purple), and calcium-coordinating amino acid positions (in orange and indicated by an asterisk). Basic residues belonging to the loops neighbouring the coordinated Ca^2+^ are reported in blue, the ones belonging to the opposite side of the domain are in turquoise. The missense mutations identified in agrin in patients affected by forms of congenital myasthenia are reported in green (see Fig. 7). Interestingly, the only β-strand that does not seem to be conserved at all is J (the 10^th^), VSGR in perlecan LG3.

>LNA1_LG1

kvavsadrdcirayqpqisstnyntltlnvktqepdnllfylgsstasdflavemrrgrv

aflwdlgsgstrlefpdfpiddnrwhsihvarfgnigslsvkemssnqksptktskspgt

anvldvnnstlmfvgglggqikkspavkvthfkgclgeaflngksiglwnyieregkcrg

c

>LNA1_LG2

dpsfhfdgsgysvvekslpatvtqiimlfntfspnglllylgsygtkdflsielfrgrvk

vmtdlgsgpitlltdrrynngtwykiafqrnrkqgvlavidayntsnketkqgetpgass

dlnrldkdpiyvgglprsrvvrrgvttksfvgciknleisrstfdllrnsygvrkgc

>LNA1_LG3

irsvsflkggyielppkslspesewlvtfattnssgiilaalggdvekrgdreeahvpff

svmliggnievhvnpgdgtglrkallhaptgtcsdgqahsislvrnrriitvqldennpv

emklgtlvesrtinvsnlyvggipegegtslltmrrsfhgciknlifnlelldfnsavgh

eqvdldtc

>LNA1_LG4

ahqfgltqnshfilpfnqsavrkklsvelsirtfassgliyymahqnqadyavlqlhggr

lhfmfdlgkgrtkvshpallsdgkwhtvktdyvkrkgfitvdgrespmvtvvgdgtmldv

eglfylgglpsqyqarkignithsipacigdvtvnskqldkdspvsaftvnrc

>LNA1_LG5

qegtyfdgsgyaalvkegykvqsdvnitlefrtssqngvllgistakvdaiglelvdgkv

lfhvnngagritaayepktatvlcdgkwhtlqankskhritlivdgnavgaesphtqsts

vdtnnpiyvggypagvkqkclrsqtsfrgclrklalikspqvqsfdfsrafelhgvflhs

c

>LNA2_LG1

kvsvssggdcirtykpeikkgsynnivvnvktavadnllfylgsakfidflaiemrkgkv

sflwdvgsgvgrveypdltiddsywyrivasrtgrngtisvraldgpkasivpsthhsts

ppgytildvdanamlfvggltgklkkadavrvitftgcmgetyfdnkpiglwnfrekegd

ckgc

>LNA2_LG2

tiqfdgegyalvsrpirwypnistvmfkfrtfsssallmylatrdlrdfmsveltdghik

vsydlgsgmasvvsnqnhndgkwksftlsriqkqanisivdidtnqeeniatsssgnnfg

ldlkaddkiyfgglptlrnlsmkarpevnlkkysgclkdieisrtpynilsspdyvgvtk

gc

>LNA2_LG3

vytvsfpkpgfvelspvpidvgteinlsfstknesgiillgsggtpapprrkrrqtgqay

yaillnrgrlevhlstgartmrkivirpepnlfhdgrehsvhvertrgiftvqvdenrry

mqnltveqpievkklfvggappefqpsplrnippfegciwnlvinsvpmdfarpvsfkna

digrc

>LNA2_LG4

skqfglsrnshiaiafddtkvknrltielevrteaesgllfymarinhadfatvqlrngl

pyfsydlgsgdthtmiptkindgqwhkikimrskqegilyvdgasnrtispkkadildvv

gmlyvgglpinyttrrigpvtysidgcvrnlhmaeapadleqptssfhvgtc

>LNA2_LG5

qrgtyfdgtgfakavggfkvgldllvefefrtttttgvllgissqkmdgmgiemideklm

fhvdngagrftavydagvpghlcdgqwhkvtankikhrieltvdgnqveaqspnpastsa

dtndpvfvggfpddlkqfglttsipfrgcirslkltkgtgkplevnfakalelrgvqpvs

c

>LNA3_LG1

pmrfngksgvevrlpndledlkgytslslflqrpnsrenggtenmfvmylgnkdasrdyi

gmavvdgqltcvynlgdreaelqvdqiltksetkeavmdrvkfqriyqfarlnytkgats

skpetpgvydmdgrnsntllnldpenvvfyvggyppdfklpsrlsfppykgcielddlne

nvlslynfkktfnlnttevepc

>LNA3_LG2

sdknyfegtgyarvptqphapiptfgqtiqttvdrgllffaengdrfislniedgklmvr

yklnselpkergvgdainngrdhsiqikigklqkrmwinvdvqntiidgevfdfstyylg

gipiairerfnistpafrgcmknlkktsgvvrlndtvgvtkkc

>LNA3_LG3

vrsasfsrggqlsftdlglpptdhlqasfgfqtfqpsgilldhqtwtrnlqvtledgyie

lstsdsggpifkspqtymdgllhyvsvisdnsglrlliddqllrnskrlkhisssrqslr

lggsnfegcisnvfvqrlslspevldltsnslkrdvslggc

>LNA3_LG4

alqfgdiptshllfklpqellkprsqfavdmqttssrglvfhtgtknsfmalylskgrlv

falgtdgkklrikskekcndgkwhtvvfghdgekgrlvvdglraregslpgnstisirap

vylgsppsgkpkslptnsfvgclknfqldskplytpsssfgvssc

>LNA3_LG5

kgiyfseegghvvlahsvllgpefklvfsirprsltgilihigsqpgkhlcvyleagkvt

asmdsgaggtstsvtpkqslcdgqwhsvavtikqhilhleldtdssytagqipfppastq

eplhlggapanlttlripvwksffgclrnihvnhipvpvtealevqgpvslngc

>LNA4_LG1

qvsmmfdgqsavevhsrtsmddlkaftslslymkppvkrpeltetadqfilylgsknakk

eymglaikndnlvyvynlgtkdveipldskpvsswpayfsivkiervgkhgkvfltvpsl

sstaeekfikkgefsgddslldldpedtvfyvggvpsnfklptslnlpgfvgclelatln

ndvislynfkhiynmdpstsvpc

>LNA4_LG2

aasyffdgsgyavvrditrrgkfgqvtrfdievrtpadnglillmvngsmffrlemrngy

lhvfydfgfsggpvhledtlkkaqindakyheisiiyhndkkmilvvdrrhvksmdnekm

kipftdiyiggappeilqsralrahlpldinfrgcmkgfqfqkkdfnlleqtetlgvgyg

c

>LNA4_LG3

srrayfngqsfiasiqkisffdgfeggfnfrtlqpngllfyyasgsdvfsisldngtvim

dvkgikvqsvdkqyndglshfvissvsptryelivdksrvgsknptkgkieqtqasekkf

yfggspisaqyanftgcisnayftrvdrdvevedfqrytekvhtslyec

>LNA4_LG4

ayqyggtansrqefehlkgdfgaksqfsirlrtrsshgmifyvsdqeendfmtlflahgr

lvymfnvghkklkirsqekyndglwhdvifirerssgrlvidglrvleeslppteatwki

kgpiylggvapgkavknvqinsiysfsgclsnlqlngasitsasqtfsvtpc

>LNA4_LG5

tgtyfsteggyvvldesfniglkfeiafevrprsssgtlvhghsvngeylnvhmkngqvi

vkvnngirdfstsvtpkqslcdgrwhritvirdsnvvqldvdsevnhvvgplnpkpidhr

epvfvggvpeslltprlapskpftgcirhfvidghpvsfskaalvsgavsinsc

>LNA5_LG1

pmkfngrsgvqlrtprdladlaaytalkfylqgpepepgqgtedrfvmymgsrqatgdym

gvslrdkkvhwvyqlgeagpavlsidedigeqfaavsldrtlqfghmsvtverqmiqetk

gdtvapgaegllnlrpddfvfyvggypstftpppllrfpgyrgciemdtlneevvslynf

ertfqldtavdrpc

>LNA5_LG2

tdgsyldgtgfarisfdsqisttkrfeqelrlvsysgvlfflkqqsqflclavqegslvl

lydfgaglkkavplqppppltsaskaiqvfllggsrkrvlvrveratvysveqdndlela

dayylggvppdqlppslrrlfptggsvrgcvkgikalgkyvdlkrlnttgvsagc

>LNA5_LG3

amtfhghgflrlalsnvapltgnvysgfgfhsaqdsallyyraspdglcqvslqqgrvsl

qllrtevktqagfadgaphyvafysnatgvwlyvddqlqqmkphrgpppelqpqpegppr

lllgglpesgtiynfsgcisnvfvqrllgpqrvfdlqqnlgsvnvstgc

>LNA5_LG4

syqfggslsshlefvgilarhrnwpslsmhvlprssrglllftarlrpgspslalflsng

hfvaqmeglgtrlraqsrqrsrpgrwhkvsvrweknrillvtdgarawsqegphrqhqga

ehpqphtlfvgglpasshssklpvtvgfsgcvkrlrlhgrplgaptrmagvtpc

>LNA5_LG5

aglffpgsggvitldlpgatlpdvglelevrplavtglifhlgqartppylqlqvtekqv

llraddgagefstsvtrpsvlcdgqwhrlavmksgnvlrlevdaqsnhtvgpllaaaaga

paplylgglpepmavqpwppaycgcmrrlavnrspvamtrsvevhgavgasgc

>SLT1_LG1

lsvnfvdrdtylqftdlqnwpranitlqvstaedngillyngdndhiavelyqghvrvsy

dpgsypssaiysaetindgqfhtvelvafdqmvnlsidggspmtmdnfgkhytlnseapl

yvggmpvdvnsaafrlwqilngtgfhgcirnlyinnelqdftktqmkpgvvpgc

>SLT2_LG1

vsvnfinkesylqipsakvrpqtnitlqiatdedsgillykgdkdhiavelyrgrvrasy

dtgshpasaiysvetindgnfhivellaldqslslsvdggnpkiitnlskqstlnfdspl

yvggmpgksnvaslrqapgqngtsfhgcirnlyinselqdfqkvpmqtgilpgc

>SLT3_LG1

itvnfvgkdsyvelasakvrpqanislqvatdkdngillykgdndplalelyqghvrlvy

dslssppttvysvetvndgqfhsvelvtlnqtlnlvvdkgtpkslgklqkqpavginspl

ylggiptstglsalrqgtdrplggfhgcihevrinnelqdfkalppqslgvspgc

>PKC_LG1

iqypqffghsyvtfeplknsyqafqitlefraeaedglllycgenehgrgdfmslaiirr

slqfrfncgtgvaiivsetkiklggwhtvmlyrdglngllqlnngtpvtgqsqgqyskit

frtplylggapsaywlvratgtnrgfqgcvqslavngrridmrpwplgkalsgadvgec

>PKC_LG2

ipqfreslrsyaatpwplepqhylsfmefeitfrpdsgdgvllysydtgskdflsinlag

ghvefrfdcgsgtgvlrsedpltlgnwhelrvsrtakngilqvdkqkivegmaeggftqi

kcntdifiggvpnyddvkknsgvlkpfsgsiqkiilndrtihvkhdftsgvnvenaahpc

>PKC_LG3

ieipqfigrsyltydnpdilkrvsgsrsnvfmrfkttakdglllwrgdspmrpnsdfisl

glrdgalvfsynlgsgvasimvngsfndgrwhrvkavrdgqsgkitvddygartgkspgm

mrqlningalyvggmkeialhtnrqymrglvgcishftlstdyhislvedavdgknintc

>PRL_LG1

vpyftqtpysflplptikdayrkfeikitfrpdsadgmllyngqkrvpgsptnlanrqpd

fisfglvggrpefrfdagsgmatirhptplalghfhtvtllrsltqgslivgdlapvngt

sqgkfqgldlneelylggypdygaipkaglssgfigcvrelriqgeeivfhdlnltahgi

shcptc

>PRL_LG2

ttpslsgagsylalpaltnthhelrldvefkplapdgvllfsggksgpvedfvslamvgg

hlefryelgsglavlrsaeplalgrwhrvsaerlnkdgslrvnggrpvlrsspgksqgln

lhtllylggvepsvplspatnmsahfrgcvgevsvngkrldltysflgsqgigqcydssp

c

>PRL_LG3

qygayfhddgflafpghvfsrslpevpetielevrtstasglllwqgvevgeagqgkdfi

slglqdghlvfryqlgsgearlvsedpindgewhrvtalregrrgsiqvdgeelvsgrsp

gpnvavnakgsvyiggapdvatltggrfssgitgcvknlvlhsarpgapppqpldlqhra

qagantrpc

>AGR_LG1

apvpafegrsflafptlrayhtlrlalefralepqglllyngnargkdflalalldgrvq

lrfdtgsgpavltsavpvepgqwhrlelsrhwrrgtlsvdgetpvlgespsgtdglnldt

dlfvggvpedqaavalertfvgaglrgcirlldvnnqrlelgigpgaatrgsgvgec

>AGR_LG2_

pfladfngfshlelrglhtfardlgekmalevvflargpsglllyngqktdgkgdfvsla

lrdrrlefrydlgkgaavirsrepvtlgawtrvslerngrkgalrvgdgprvlgespvph

tvlnlkeplyvggapdfsklaraaavssgfdgaiqlvslggrqlltpehvlrqvdvtsfa

ghpc

>AGR_LG3

navteselaneipvpetldsgalhekalqsnhfelslrteatqglvlwsgkateradyva

laivdghlqlsynlgsqpvvlrstvpvntnrwlrvvahreqregslqvgneapvtgsspl

gatqldtdgalwlgglpelpvgpalpkaygtgfvgclrdvvvgrhplhlledavtkpelr

pc

>NRX1A_LG1

glefpgaegqwtrfpkwnaccesemsfqlktrsarglvlyfddegfcdfleliltrggrl

qlsfsifcaepatlladtpvndgawhsvrirrqfrnttlfidqveakwvevkskrrdmtv

fsglfvgglppelraaalkltlasvrerepfkgwirdvrvnssqvlpvdsgevklddepp

nsgggspc

>NRX1A_LG2

iatfkgseyfcydlsqnpiqsssdeitlsfktlqrnglmlhtgksadyvnlalkngavsl

vinlgsgafealvepvngkfndnawhdvkvtrnlrqvtisvdgiltttgytqedytmlgs

ddffyvggspstadlpgspvsnnfmgclkevvyknndvrlelsrlakqgdpkmkihgvva

fkc

>NRX1A_LG3

dpitfetpesfislpkwnakktgsisfdfrttepnglilfshgkprhqkdakhpqmikvd

ffaiemldghlyllldmgsgtikikallkkvndgewyhvdfqrdgrsgtisvntlrtpyt

apgeseildlddelylgglpenkaglvfptevwtallnygyvgcirdlfidgqskdirqm

aevqstagvkpsc

>NRX1A_LG4

vlsydgsmfmkiqlpvvmhteaedvslrfrsqraygilmattsrdsadtlrleldagrvk

ltvnldcirincnsskgpetlfagynlndnewhtvrvvrrgkslkltvddqqamtgqmag

dhtrlefhnietgiiterrylssvpsnfighlqsltfngmayidlckngdidyc

>NRX1A_LG5

dpvtfktkssyvalatlqaytsmhlffqfkttsldglilynsgdgndfivvelvkgylhy

vfdlgnganlikgssnkplndnqwhnvmisrdtsnlhtvkidtkittqitagarnldlks

dlyiggvaketykslpklvhakegfqgclasvdlngrlpdlisdalfcngqiergc

>NRX1A_LG6

yifskgggqitykwppndrpstradrlaigfstvqkeavlvrvdsssglgdylelhihqg

kigvkfnvgtddiaieesnaiindgkyhvvrftrsggnatlqvdswpvierypagrqlti

fnsqatiiiggkeqgqpfqgqlsglyynglkvlnmaaendaniaivgnv

>NRX2A_LG1

glefgggpgqwaryarwagaassgelsfslrtnatralllylddggdcdflelllvdgrl

rlrftlscaepatlqldtpvaddrwhmvlltrdarrtalavdgearaaevrskrremqva

sdlfvggippdvrlsaltlstvkyeppfrgllanlklgerppallgsqglrgatadplc

>NRX2A_LG2

vatfkgneffcydlshnpiqsstdeitlafrtlqrnglmlhtgksadyvnlslksgavwl

vinlgsgafealvepvngkfndnawhdvrvtrnlrqhagighamvnklhylvtisvdgil

tttgytqedytmlgsddffyiggspntadlpgspvsnnfmgclkdvvyknndfklelsrl

akegdpkmklqgdlsfrc

>NRX2A_LG3

dpvtfespeafvalprwsakrtgsisldfrttepnglllfsqgrragggagshssaqrad

yfamelldghlyllldmgsggiklrassrkvndgewchvdfqrdgrkgsisvnsrstpfl

atgdseildleselylgglpeggrvdlplppevwtaalragyvgcvrdlfidgrsrdlrg

laeaqgavgvapfc

>NRX2A_LG4

vlsydgsmymkimlpnamhteaedvslrfmsqrayglmmattsresadtlrleldggqmk

ltvnldclrvgcapskgpetlfaghklndnewhtvrvvrrgkslqlsvdnvtvegqmaga

hmrlefhnietgimterrfisvvpsnfighlsglvfngqpymdqckdgdityc

>NRX2A_LG5

dpvtfksrssylalatlqayasmhlffqfkttapdglllfnsgngndfivielvkgyihy

vfdlgngpslmkgnsdkpvndnqwhnvvvsrdpgnvhtlkidsrtvtqhsngarnldlkg

elyigglsknmfsnlpklvasrdgfqgclasvdlngrlpdliadalhrigqvergc

>NRX2A_LG6

ttyifgkggalitytwppndrpstrmdrlavgfsthqrsavlvrvdsasglgdylqlhid

qgtvgvifnvgtdditidepnaivsdgkyhvvrftrsggnatlqvdswpvnerypagnfd

nerlaiarqripyrlgrvvdewlldkgrqltifnsqaaikiggrdqgrpfqgqvsglyyn

glkvlalaaesdpnvrteghlrlvgegps

>NRX3A_LG1

glefmglpnqwarylrwdastrsdlsfqfktnvstglllylddggvcdflclslvdgrvq

lrfsmdcaetavlsnkqvndsswhflmvsrdrlrtvlmldgegqsgelqpqrpymdvvsd

lflggvptdirpsaltldgvqampgfkglildlkygnseprllgsrgvqmdaegpc

>NRX3A_LG2

vatfrgseylcydlsqnpiqsssdeitlsfktwqrnglilhtgksadyvnlalkdgavsl

vinlgsgafeaivepvngkfndnawhdvkvtrnlrqvtisvdgiltttgytqedytmlgs

ddffyvggspstadlpgspvsnnfmgclkevvyknndirlelsrlariadtkmkiygevv

fkc

>NRX3A_LG3

dpinfetpeayislpkwntkrmgsisfdfrttepnglilfthgkpqerkdarsqkntkvd

ffavelldgnlyllldmgsgtikvkatqkkandgewyhvdiqrdgrsgtisvnsrrtpft

asgeseildlegdmylgglpenraglilptelwtamlnygyvgcirdlfidgrsknirql

aemqnaagvkssc

>NRX3A_LG4

ilsydgsmymkiimpmvmhteaedvsfrfmsqraygllvattsrdsadtlrleldggrvk

lmvnldcirincnsskgpetlyagqklndnewhtvrvvrrgkslkltvdddvaegtmvgd

htrlefhnietgimtekryisvvpssfighlqslmfngllyidlckngdidyc

>NRX3A_LG5

dpvtfktkssylslatlqaytsmhlffqfkttspdgfilfnsgdgndfiavelvkgyihy

vfdlgngpnvikgnsdrplndnqwhnvvitrdnsnthslkvdtkvvtqvingaknldlkg

dlymaglaqgmysnlpklvasrdgfqgclasvdlngrlpdlindalhrsgqiergc

>NRX3A_LG6

atyifgksgglilytwpandrpstrsdrlavgfsttvkdgilvridsapglgdflqlhie

qgkigvvfnigtvdisikeertpvndgkyhvvrftrnggnatlqvdnwpvnehyptgrql

tifntqaqiaiggkdkgrlfqgqlsglyydglkvlnmaaennpnikingsv

>NRX1B_LG1

yifskgggqitykwppndrpstradrlaigfstvqkeavlvrvdsssglgdylelhihqg

kigvkfnvgtddiaieesnaiindgkyhvvrftrsggnatlqvdswpvierypagrqlti

fnsqatiiiggkeqgqpfqgqlsglyynglkvlnmaaendaniaivgnv

>NRX2B_LG1

ttyifgkggalitytwppndrpstrmdrlavgfsthqrsavlvrvdsasglgdylqlhid

qgtvgvifnvgtdditidepnaivsdgkyhvvrftrsggnatlqvdswpvnerypagnfd

nerlaiarqripyrlgrvvdewlldkgrqltifnsqaaikiggrdqgrpfqgqvsglyyn

glkvlalaaesdpnvrteghlrlvgegps

>NRX3B_LG1

atyifgksgglilytwpandrpstrsdrlavgfsttvkdgilvridsapglgdflqlhie

qgkigvvfnigtvdisikeertpvndgkyhvvrftrnggnatlqvdnwpvnehyptgrql

tifntqaqiaiggkdkgrlfqgqlsglyydglkvlnmaaennpnikingsv

**Figure S2: Sequences of human LG domains.** All the sequences are reported in FASTA format. The accession codes of the corresponding proteins and proteoglycans are reported in Table 1.

**Table S1**

**Frequency of basic and acidic residues within the LG-domains belonging to human proteins that interact with α-dystroglycan**

**Protein (Arg + Lys)(%) (Asp + Glu)(%)**

Laminin-α1* 11.2 9.0
Laminin-α2* 11.8 10.3
Laminin-α3* 10.9 9.4
Laminin-α4* 11.0 11.2
Laminin-α5* 9.6 7.9

Slit-1 4.6 10.9
Slit-2 8.0 9.1
Slit-3 8.0 9.1

Pikachurin* 10.6 9.5

Perlecan* 8.7 9.3

Agrin* 10.1 11.0

Neurexin1α* 10.5 11.4
Neurexin2α* 10.5 11.2
Neurexin3α* 10.2 11.4

Neurexin1β* 9.4 9.4
Neurexin2β* 11.0 10.5
Neurexin3β* 10.6 8.7

*average value from multiple LG domains. No significant deviation from the average values were observed with some few notable exceptions: LNA3’s LG4 (Basic: 13.9, Acidic: 6.6), LNA5’s LG4 (Basic: 13.8, Acidic: 3.5), SLT1’s LG1 (Basic 4.6, Acidic: 10.9).
